# Current state of the art and future directions for implantable sensors in medical technology: Clinical needs and engineering challenges

**DOI:** 10.1063/5.0152290

**Published:** 2023-09-27

**Authors:** David Yogev, Tomer Goldberg, Amir Arami, Shai Tejman-Yarden, Thomas E. Winkler, Ben M. Maoz

**Affiliations:** 1 The Engineering Medical Research Lab, Sheba Medical Center, Ramat Gan, Israel; 2 Faculty of Medicine, School of Medicine, Tel Aviv University, Tel Aviv 69978, Israel; 3 Hand Surgery Department, Microsurgery, and Peripheral Nerve Surgery Unit, Sheba Medical Center, 52621 Tel Hashomer, Israel; 4 Institute of Microtechnology, Technische Universität Braunschweig, 38106 Braunschweig, Germany; 5 Center of Pharmaceutical Engineering, Technische Universität Braunschweig, 38106 Braunschweig, Germany; 6 Department of Biomedical Engineering, Tel Aviv University, Tel Aviv 69978, Israel; 7 Sagol School of Neuroscience, Tel Aviv University, Tel Aviv 69978, Israel; 8 The Center for Nanoscience and Nanotechnology, Tel Aviv University, Tel Aviv 69978, Israel; 9 Sagol Center for Regenerative Medicine, Tel Aviv University, Tel Aviv 69978, Israel

## Abstract

Implantable sensors have revolutionized the way we monitor biophysical and biochemical parameters by enabling real-time closed-loop intervention or therapy. These technologies align with the new era of healthcare known as healthcare 5.0, which encompasses smart disease control and detection, virtual care, intelligent health management, smart monitoring, and decision-making. This review explores the diverse biomedical applications of implantable temperature, mechanical, electrophysiological, optical, and electrochemical sensors. We delve into the engineering principles that serve as the foundation for their development. We also address the challenges faced by researchers and designers in bridging the gap between implantable sensor research and their clinical adoption by emphasizing the importance of careful consideration of clinical requirements and engineering challenges. We highlight the need for future research to explore issues such as long-term performance, biocompatibility, and power sources, as well as the potential for implantable sensors to transform healthcare across multiple disciplines. It is evident that implantable sensors have immense potential in the field of medical technology. However, the gap between research and clinical adoption remains wide, and there are still major obstacles to overcome before they can become a widely adopted part of medical practice.

## I. INTRODUCTION

The development of implanted sensors has been driven by the demand for more precise physiological recordings specific to the circumstances in the vicinity of a clinically relevant physiological event.^1^ Ideally, these can even provide a real-time closed-loop intervention or therapy. Today, implantable sensors can provide accurate *in vivo* measurements of biophysical and bioelectrical parameters, as well as specific biomarkers such as ions (e.g., Na^+^, K^+^, and Ca^2+^), neurotransmitters (e.g., serotonin, epinephrine, norepinephrine, serotonin, and dopamine), hormones (e.g., melatonin and cortisol), and volatile organic compounds (e.g., acetone, isopropyl alcohol, and isoprene).^2^

Engineers and clinicians can now implant increasingly smaller devices inside the human body, thanks to ongoing advances in microfabrication technology.^3^ For example, a modern pacemaker can be as small as 1 in. (2.5 cm) and weigh less than 15 g.^3,4^ Continuous artery monitoring is another example of the revolutionary potential of implanted sensors. This can be accomplished by implanting a sensor during surgery that is wirelessly monitored by patients after surgery. These sensors can contribute to the early detection and prevention of a variety of conditions, such as limb ischemia, strokes, myocardial infarctions, early stenosis, decreased blood flow, and clots.^5^

The promise of implanted sensors, however, extends far beyond cardiovascular disorders. With thousands of people who have already benefited from implanted deep brain stimulators (DBS),^6,7^ smart ingestible pills for precise tracking when a medicine is administered,^8^ and even implantable tools designed to restore damaged nerves,^9,10^ healthcare is approaching a new era known as Healthcare 5.0. Compared to Healthcare 4.0, which focused on integrating digital technologies with healthcare services,^11^ the era of Healthcare 5.0 will herald smart disease control and detection, virtual care, intelligent health management, smart monitoring, and decision-making.^11^ Implantable sensors will play a vital role in this paradigm shift by enabling continuous physiological monitoring at a high level of accuracy, which is likely to contribute to greater success in integrating advanced technologies such as artificial intelligence and the Internet of Things with medical devices and treatment.

Ideally, implantable sensors should be precise, reliable, stable over the long-term, with minimal fouling or drift, and be sensitive and resilient to mechanical forces in an often hostile environment.^12,13^ The need for calibration, power dissipation, thermal stress,^14^ and the security of the data,^15^ which must be protected under limited power and computing resources, are all major challenges to the design of sensors and constitute today's cutting-edge research topics.

**FIG. 1.**
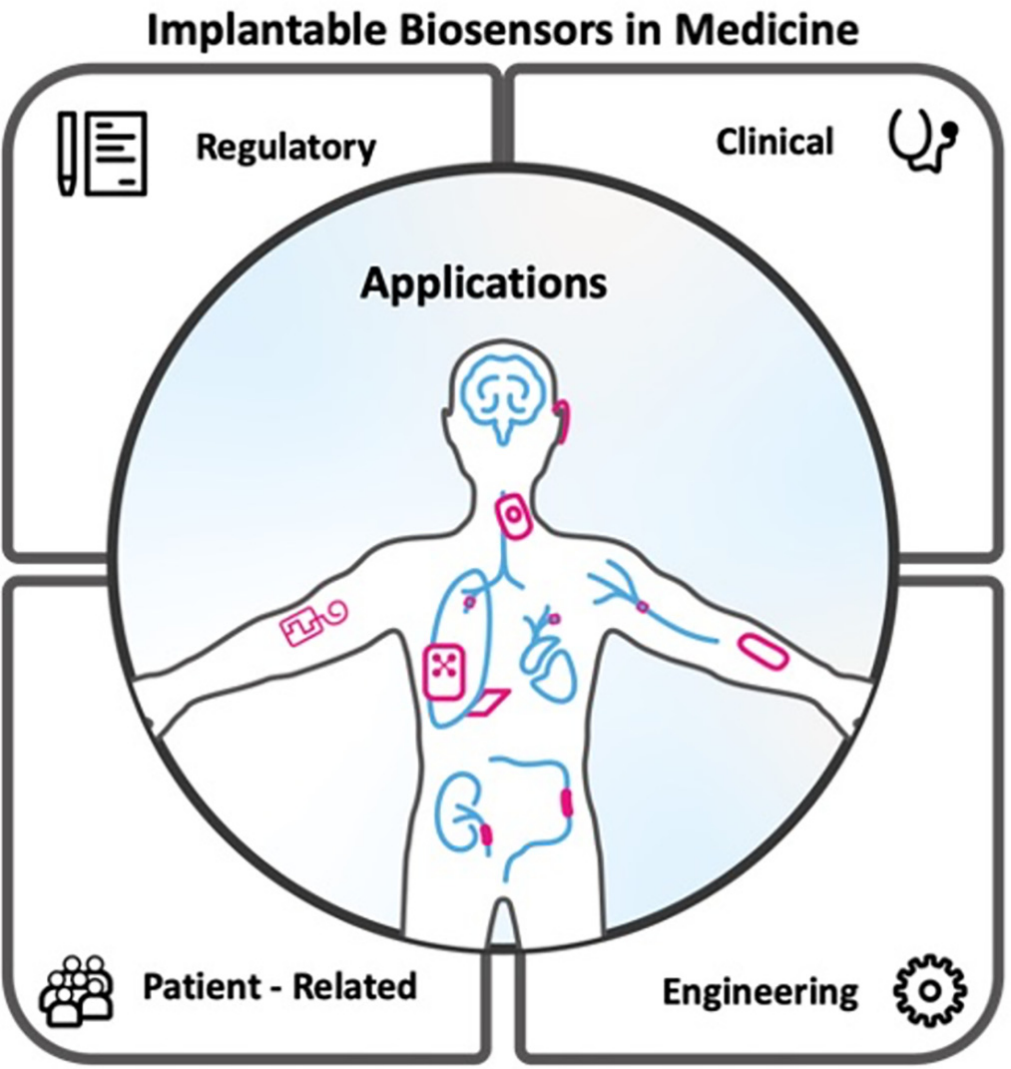
The four main types of challenges in the development and implementation of implantable sensors and their different types of applications.

This review aims to provide a comprehensive overview of the integration of implantable sensors in a wide range of medical application fields, by highlighting the challenges to their adoption (Fig. 1). First, we examine the advantages and disadvantages of implantable sensors by contrasting them with the similarly growing wearable sensor field. We then look at the different types of implantable temperature, mechanical, electrical, electrochemical, and optical sensors. We present examples of these sensors and systems that have been used in cardiology, pulmonology, neurology, gastroenterology, urology, orthopedics, and otolaryngology. The overarching goal of this review is to better understand the engineering, technical, and clinical hurdles that currently curtail the clinical adoption of implantable sensors, including lack of information and high costs, patient-related issues such as discomfort and social norms, and regulatory challenges.

We aim to present a concise and informative reference for researchers, clinicians, and policymakers from a broad range of backgrounds who are interested in integrating implantable sensors into medical practice. We stress the gaps between sensor research and clinical adoption and point out the major stumbling blocks that need to be overcome for implantable sensors to become more accessible and integrated into today's clinical practice.

## II. IMPLANTABLE VS WEARABLE SENSORS: DEVELOPMENT CHALLENGES

In recent years, there have been significant advances in the development and fabrication of sensors, leading to a rise in publications, companies, and clinical trials that utilize these sensors.^1,2^ The increasing interest and growth in this field are evident in the remarkable number of review papers.^3,4^ A closer look at this trend reveals that different types of sensors have developed at different rates, with the implantable sensor market valued at almost 15 times the global wearable sensor market, which was valued at USD 327.68 million in 2021. However, the projected growth rate of the wearable sensors market is much larger, with a compound annual growth rate (CAGR) of 18.3% for the 2022–2030 forecast period, compared to a CAGR of 11.1% for the implantable sensor market from 2020 to 2027.^16,17^

**FIG. 2.**
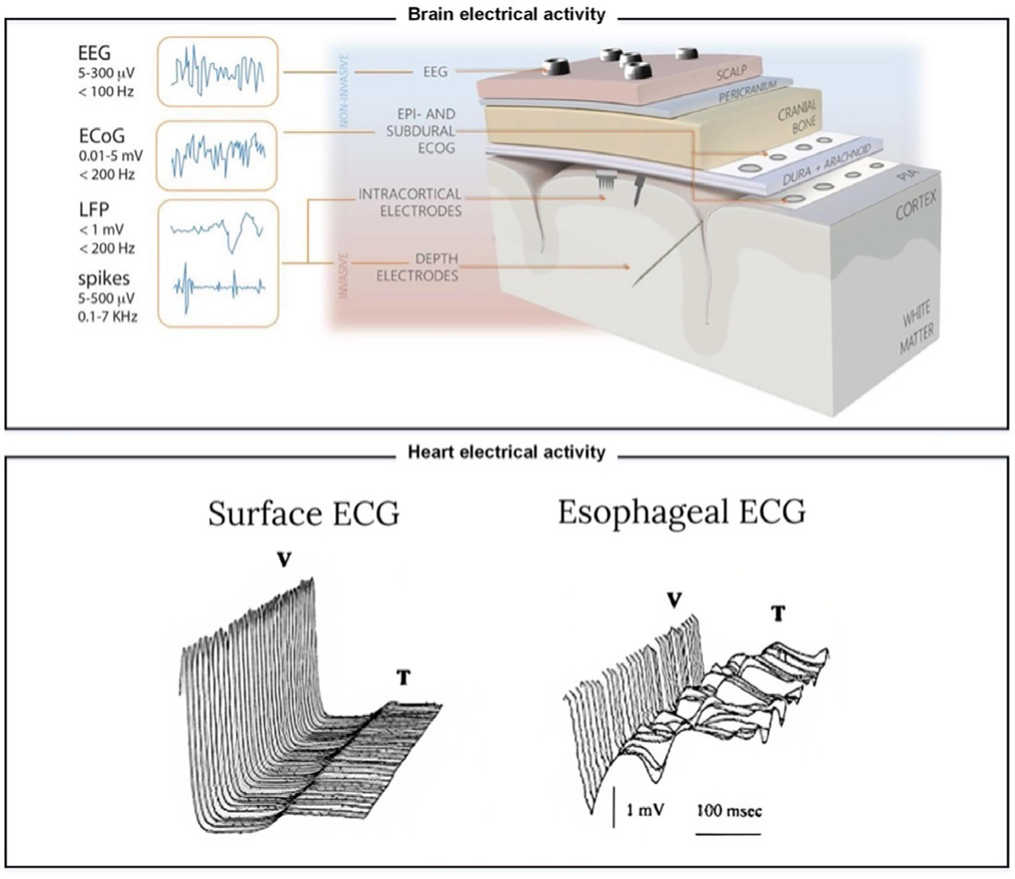
Illustration of electrode types for electrophysiological recordings of brain and heart activity. It illustrates the importance of proximity in accurately recording brain and heart electrical activity and complements Table I. The top panel displays different types of electrodes for recording brain activity. It shows that implantable and invasive sensors allow for more accurate recordings as they get closer to the brain tissue. Reproduced with permission from Cutrone *et al.*, Adv. Healthcare Mater. **8**, 1801345 (2019). Copyright 2019 John Wiley and Sons.^9^ Reproduced with permission from Szostak *et al.*, Front. Neurosci. **11**, 665J (2017). Copyright 2017 Authors, licensed under a Creative Commons (CC BY) license.^46^ The bottom panel demonstrates the importance of proximity in recording heart activity. Signals during myocardial ischemia were recorded using surface ECG (external sensor) and esophageal ECG (closer to the heart).^35^ It shows that signals closer to the heart are more accurate than those recorded externally. Adapted with permission from Mächler *et al.*, Internet J. Thorac. Cardiovasc. Surg. **2**, 8758 (1998). Copyright 1998 ISPUB, www.ispub.com.^35^

Although wearable sensors are not the focus of this review, it is important to understand the differences between the two to better grasp the challenges related to developing and implementing implantable sensors. Noninvasive, wearable sensors (such as smartwatches) are designed to be convenient ergonomically and functionally, fashionable, appealing, and easy to use. These sensors allow easy access to real-time data and patient self-monitoring. However, because patients can remove these wearables at will, they require compliance and maintenance to avoid loss or damage. These sensors are also limited to bio-signals that can be extrapolated from skin measurements such as surface electrocardiogram (ECG), blood oxygen saturation, skin temperature, and blood pressure.^13,18^ These measurements can often be approximations or manipulations of sampled raw data and thus may not be as accurate as implantable sensors.^19,20^ In Table I and Fig. 2, we provide a comparison of signals that can be recorded by both implantable sensors and wearable sensors for various biological parameters and highlight the differences in sensitivity and accuracy between the two types of sensors.

**TABLE I.** Comparison of biological signals that can be measured at different depths inside the human body by implantable and wearable sensors. Each example was sourced from a single study, and no quantitative methods were used to synthesize or pool the information.

(A) Electrophysiology—brain electrical activity.^a^			
Comparison	What is measured?	Amplitude	Frequencies
Electroencephalogram (EEG; on top of scalp)	EEG	5–300 *μ*V	<100 Hz
Epidural and subdural electrocorticography (ECoG)	ECoG	0.01–5 mV	<200 Hz
Intracortical electrodes	Local field potentials (LFP)	<1 mV	<200 Hz
Depth electrodes	Spikes	5–500 *μ*V	0.1–7 Hz

(B) Electrophysiology—heart electrical activity (Refs. 34 and 35).^b^				
Method	Accuracy	Description	Detection of ischemic episodes	Detection of arrhythmia episodes
Standard surface electrocardiogram (S-ECG)	Moderate	Recording of the heart's electrical activity from electrodes placed on the skin of the chest, arms, and legs.	Compared to surface ECG measurements, esophageal ECG showed a significant improvement of 46%–67% in detection	Compared to surface ECG measurements, esophageal ECG showed a significant improvement of 52% in detection
Esophageal (E-ECG)	High	Recording of the heart's electrical activity from a small electrode placed inside the esophagus, close to the heart. It provides a closer look at the heart's activity than a standard ECG.		

(C) Vital signs—blood oxygen levels (oxygen saturation).^c^			
Method	Bias (%)	Precision (SD)	Ability to obtain successful readings
Pulse oximetry	<1	1.0%–1.2%	59%–84%
Arterial catheter oximetry	<1	0.5%–1.0%	99%–100%

(D) Vital signs—blood pressure (BP) monitoring (Refs. 37–41).^d^		
Measurement	Observations	Accuracy
Cuff-based BP measurement	Underestimated intra-arterial brachial systolic BP	Inaccuracy of −5.7 mm Hg
	Overestimated intra-arterial diastolic BP	Inaccuracy of 5.5 mm Hg
Cuff-less photoplethysmography-based wearable device	96.31% agreement in identifying hypertension and an interclass correlation coefficient of 0.99 and 0.97 for systolic and diastolic measurements, respectively, compared to cuff-based measurement	An earlier review and meta-analysis found that the available data are still insufficient to assess the reliability of noninvasive blood pressure recording devices
Invasive BP monitoring via peripheral artery catheterization	Preferred in situations where there may be significant hemodynamic changes, frequent blood sampling is required, or continuous and precise beat-to-beat BP monitoring is necessary	The reliability and accuracy of this system are unsurpassed, particularly when anticipating fluctuations in BP^24^

(E) Biomolecular measurements—glucose monitoring (Refs. 42–45).^e^				
Comparison	What is measured?	Time delay	Mean absolute relative difference (MARD^f^) compared to reference glucose values	Ability to measure time-in-range^g^ (considered the new gold standard for long-term glucose monitoring)
Traditional finger stick testing	Capillary blood glucose	Minimal	None	Not available
Wearable continuous glucose monitoring	Interstitial fluid glucose	Physiological time delay (during exercise and/or after a meal)	9.6–32.1%	Available
Implantable continuous glucose monitoring (Eversense^®^)	Interstitial fluid glucose	Physiological time delay (during exercise and/or after a meal)	8.8%–11.6%	Available
Implantable continuous glucose monitoring (future system^h^)	Capillary blood glucose at the level of internal organs, venous blood, arterial blood	Minimal	None	If developed, will be more accurate

^a^ Summarizes different electrophysiology techniques used to measure brain electrical activity, including electroencephalogram (EEG), epidural and subdural electrocorticography (ECoG), local field potentials (LFP), and depth electrodes (illustrated in Fig. 2). The metrics include amplitude and frequencies of brain electrical activity recorded by each technique. Each technique has its own advantages and disadvantages, and selecting the appropriate technique depends on the research question and the spatial and temporal resolution required. Adapted with permission from Cutrone *et al.*, Adv. Healthcare Mater. **8**, 1801345 (2019). Copyright 2019 John Wiley and Sons.^9^

^b^ Compares two methods of measuring heart electrical activity: the standard surface electrocardiogram (S-ECG) and esophageal electrocardiogram (E-ECG). The E-ECG is more accurate than the S-ECG in detecting both ischemic and arrhythmia episodes. Exemplary recordings are shown in Fig. 2.

^c^ Compares two methods of measuring blood oxygen levels: arterial catheter oximetry (implantable) and pulse oximetry (wearable). Both methods are accurate, but arterial catheter oximetry has a higher success rate in obtaining readings and slightly better precision than pulse oximetry. Adapted with permission from Haessler *et al.*, J. Cardiothorac. Vasc. Anesth. **6**, 668–673 (1992). Copyright 1992 Elsevier.^36^ Bias refers to the average difference between the measured values and the values obtained by immediately analyzing arterial blood samples on a calibrated IL 282 co-oximeter (Instrumentation, Lexington, MA). Precision (SD) refers to the standard deviation of the measured values compared to the values obtained by CO-oximetry.

^d^ Compares three methods of measuring blood pressure: cuff-based measurement, cuff-less photoplethysmography-based wearable devices, and invasive BP monitoring via peripheral artery catheterization. Cuff-based measurement can be inaccurate, whereas cuff-less photoplethysmography-based wearable devices are a convenient and accurate alternative. However, invasive BP monitoring is the most reliable and accurate method, especially in situations where precise monitoring is necessary.

^e^ Compares traditional finger stick testing with two continuous glucose monitoring methods: wearable and implantable devices. Implantable continuous glucose monitoring shows promise for accurately measuring glucose levels with minimal time delays and is capable of measuring time-in-range. The future system is a hypothetical glucose monitoring system that combines currently available technologies for optimal accuracy. The mean absolute relative difference (MARD) is used to compare the accuracy of the methods.

^f^ MARD (%) = [|Continuous monitor glucose − Glucose meter glucose|/(Glucose meter glucose)] × 100%.

^g^ Time-in-range is the amount of time in % that the person's blood glucose remains within the target range [70–180 mg/dl (3.9–10.0 mmol/l)], measured by real-time continuous glucose monitoring (rtCGM) and intermittently viewed CGM (iCGM) devices.

^h^ This future system is a hypothetical glucose monitoring system that represents the ideal combination of currently available technologies. It is presented here for illustrative purposes only and is not an actual system available on the market.

On the other hand, implantable sensors can be used for long-term monitoring aimed at significant long-term physiological evaluations that give an accurate and detailed view of the body's internal environment. These sensors offer real-time data collection and monitoring, which can help detect the early onset of anomalies and complications.^1^ In addition, these sensors can be integrated with other implantable or wearable devices, allowing for the delivery of treatment such as electrical pulses or drugs in a controlled, targeted manner.^21^ However, implantable sensors, unlike wearable sensors, are invasive and require surgical insertion, which is considerably more expensive and entails possible risk of infection or rejection by the body, in addition to calling for specialized insertion, replacement, or removal training, thus making this approach beyond the reach of today's everyday practices.^22^

It is worth noting that in addition to implantable and wearable sensors, some devices can seamlessly integrate implantable and wearable technologies. The best examples of this type of integration are implantable neuroprosthetics that replace motor, sensory, or cognitive functions impaired by injury or disease. Their integration forms a versatile sensor platform that partially resides within the body but is connected to a wearable external unit to produce a comprehensive solution that reinstates compromised or altered functions.^23,24^ This “third” intriguing category of sensors includes intelligent prostheses (artificial limbs) that can be controlled by the brain,^25,26^ e-skin that transmits sensations to the brain,^27^ cochlear implants that stimulate the auditory nerve to improve hearing,^28^ and retinal implants (visual prosthetics), which stimulate nerves in the visual system and enable individuals with visual impairments to perceive light and recognize objects.^29^

While this is an important growing field, it is beyond the scope of this paper. To delve into the topic further, interested readers can refer to recent review papers in this field.^9,24,30–33^

## III. TYPES AND APPLICATIONS OF IMPLANTABLE SENSORS: AN OVERVIEW

Implantable sensors can be classified in multiple ways, and each classification method can provide insights and information on different aspects of their actions and mechanisms. Table II presents several of these categorization methods.

**TABLE II.** Categorization methods for implantable sensors.^47–50^

Categorization method	Examples	Information provided by this categorization method
Sensing mechanism and biomarker	Temperature, pressure, amperometric or optical glucose, etc. (see Sec. III A and Table III)	Comparison of analytical performance, optimization of engineering aspects
Application area	Blood pressure monitoring, arrhythmia detection, diabetes management, etc. (see Sec. III B and Table IV)	Clinical utility, potential impact, and the intended duration of use of the sensors (i.e., acute invasive diagnostics versus chronic implantation)
Durability	Weeks (wound healing) to months (biochemical sensors) to years (biophysical sensors) (cf. Secs. IV C and IV G)	Long-term cost-effectiveness
Implantation approach	Subcutaneous, endovascular, deep tissue surgery, etc. (cf. Sec. IV D)	Surgical technique, equipment needed, safety profile, potential risks, the proximity of the sensor to the target organ
Communication and energy	Wireless radio frequency transmission, ultrasonic links, batteries, power harvesters, etc. (cf. Secs. IV E and IV F)	Potential for interference, best site for implantation

Arterial lines are a good example of these classification schemes. Arterial catheterization (or arterial line insertion)^51^ is frequently performed in operating rooms and intensive care units. Through the cannulation of a peripheral artery, arterial lines enable regular arterial blood collection and continuous monitoring of the patient's blood pressure.^51^ They also provide an indirect measurement of cardiac output.^52^ The pressure sensing system employs a transducer to detect pressure changes inside the artery. The transducer converts these changes into electrical signals that are then transmitted to a monitor for display and analysis.^53^ Arterial lines, with their diverse implantation methods and established routine clinical use for acute invasive diagnostics can thus be variously classified by biomarker (blood pressure and cardiac function), by the working principle or sensing mechanism (fluid wave translated into an electronic signal), and/or by their duration of use (acute invasive care).

In this review paper, we focus on two approaches: sensor classification by their application area and classification by working principle. This dual perspective is aimed at providing maximal insights into their clinical utility and potential impact on healthcare.

### A. Types of implantable sensors

Implantable sensors are devices that are placed in the body to measure various biological parameters or biomarkers. In the following, we start with a broad conceptual overview of the range of available sensing approaches. To do so, we classify them in terms of the biomarker and the sensor's working principle (which in many cases are related).

The first major group is sensors aimed at biophysical biomarkers, including temperature, motion, stress/strain and pressure, electrophysiology, and bioimpedance. These sensors all rely on well-established measurement principles, resulting in miniaturized devices that are still relatively simple and robust. They also have low power requirements, except in electrophysiology (where, especially in the brain, weak signals can require advanced amplifiers) and bioimpedance (requiring comparatively complex readout). Their typical disadvantages include their sensitivity to noise from other sources that also produce similar signals (e.g., external motion and external electric fields). Mechanical sensors are often cross-sensitive to temperature fluctuation.

The second major group is sensors aimed at biochemical biomarkers, which we further categorize into potentiometric, amperometric, optical, and affinity-based (bio)sensors based on their working principle. These sensors are generally capable of achieving high sensitivity and specificity, though interference from much-higher-concentration species can still pose challenges. The need for chemical sensitivity and specificity requires more complex and often more fragile construction, resulting in shorter service lifetimes.

**FIG. 3.**
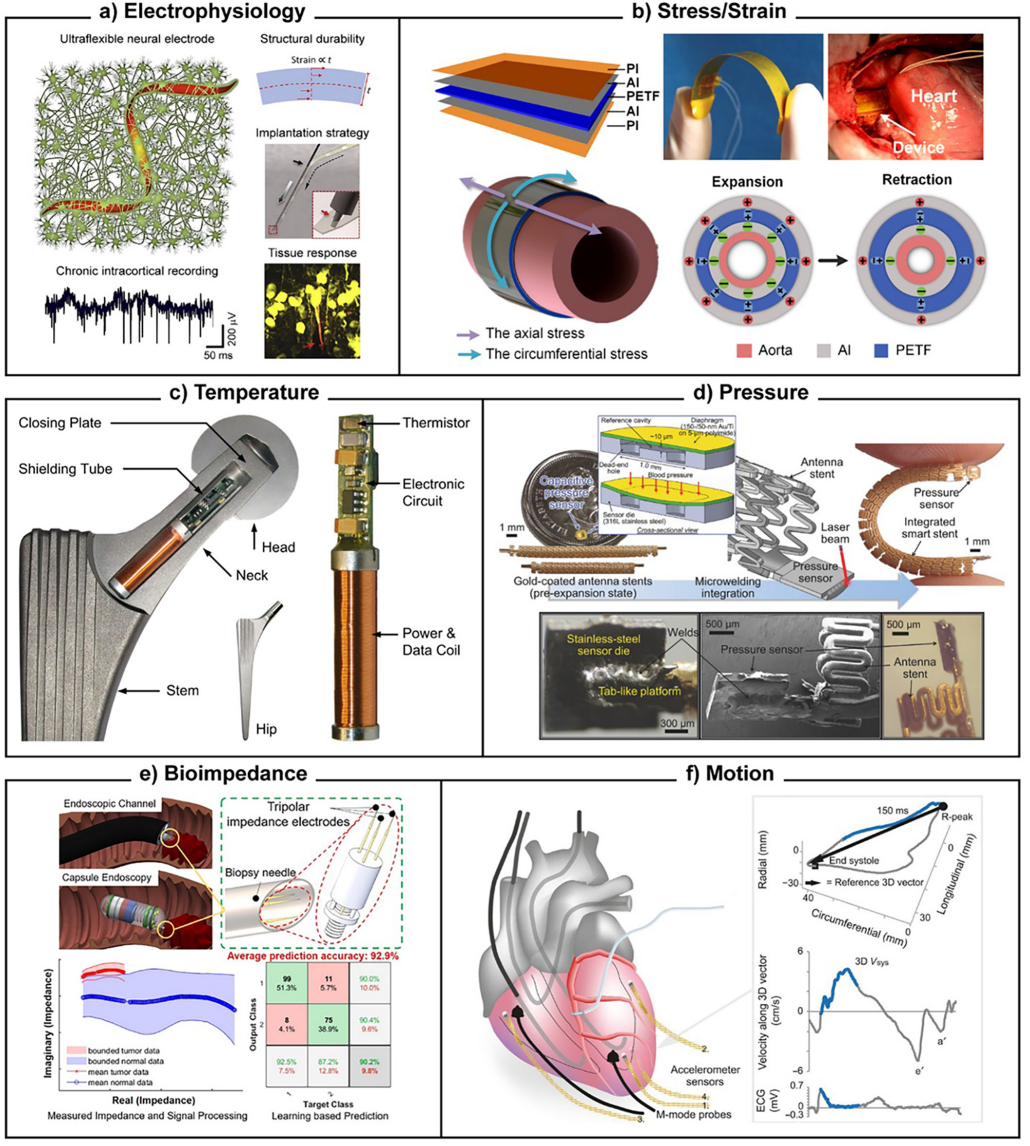
Implantable sensor examples for biophysical parameters. (a) Electrophysiology: flexible neural probes record extracellular neuronal potentials with minimal tissue disruption. The figure shows the typical sensor concept, readout, and design considerations. Reproduced with permission from He *et al.*, iScience **23**, 101387 (2020). Copyright 2020 Elsevier.^67^ (b) Stress/strain: a blood pressure sensor based on monitoring arterial wall strain using a piezoelectric cuff wrapped around the artery. The figure illustrates structure, function, and implantation. Reproduced with permission from Cheng *et al.*, Nano Energy **22**, 453–460 (2016). Copyright 2016 Elsevier.^63^ (c) Temperature: this sensor is intended to monitor hip implant failure from friction-induced heating. The figure shows its placement and various sensor package components. Reproduced with permission from PLoS One **7**, e43489 (2012). Copyright 2012 Authors, licensed under a Creative Commons (CC BY) license.^56^ (d) Pressure: another blood pressure sensor, with a stent-mounted package directly monitoring liquid pressure. The figure illustrates the sensing principle as well as various schematics and views of package components. Reproduced with permission from Chen *et al.*, Adv. Sci. **5**, 1700560 (2018). Copyright 2018 Authors, licensed under a Creative Commons (CC BY) license.^64^ (e) Bioimpedance: a sensor designed for a GI capsule endoscopy (or traditional endoscope) to detect colorectal tumor tissue. The figure illustrates mounting concepts as well as sensor performance. Reproduced with permission from Nguyen *et al.*, ACS Sens. **7**, 632–640 (2022). Copyright 2022 ACS.^72^ (f) Motion: MEMS accelerometers mounted on the heart to monitor muscle motion. The figure illustrates sensor placement (alongside additional probes for comparison), and also compares sensor output to traditional ECG. Reproduced with permission from Grymyr *et al.*, Interact. Cardiovasc. Thorac. Surg. **21**, 573–582 (2015). Copyright 2015 OUP.^57^

**FIG. 4.**
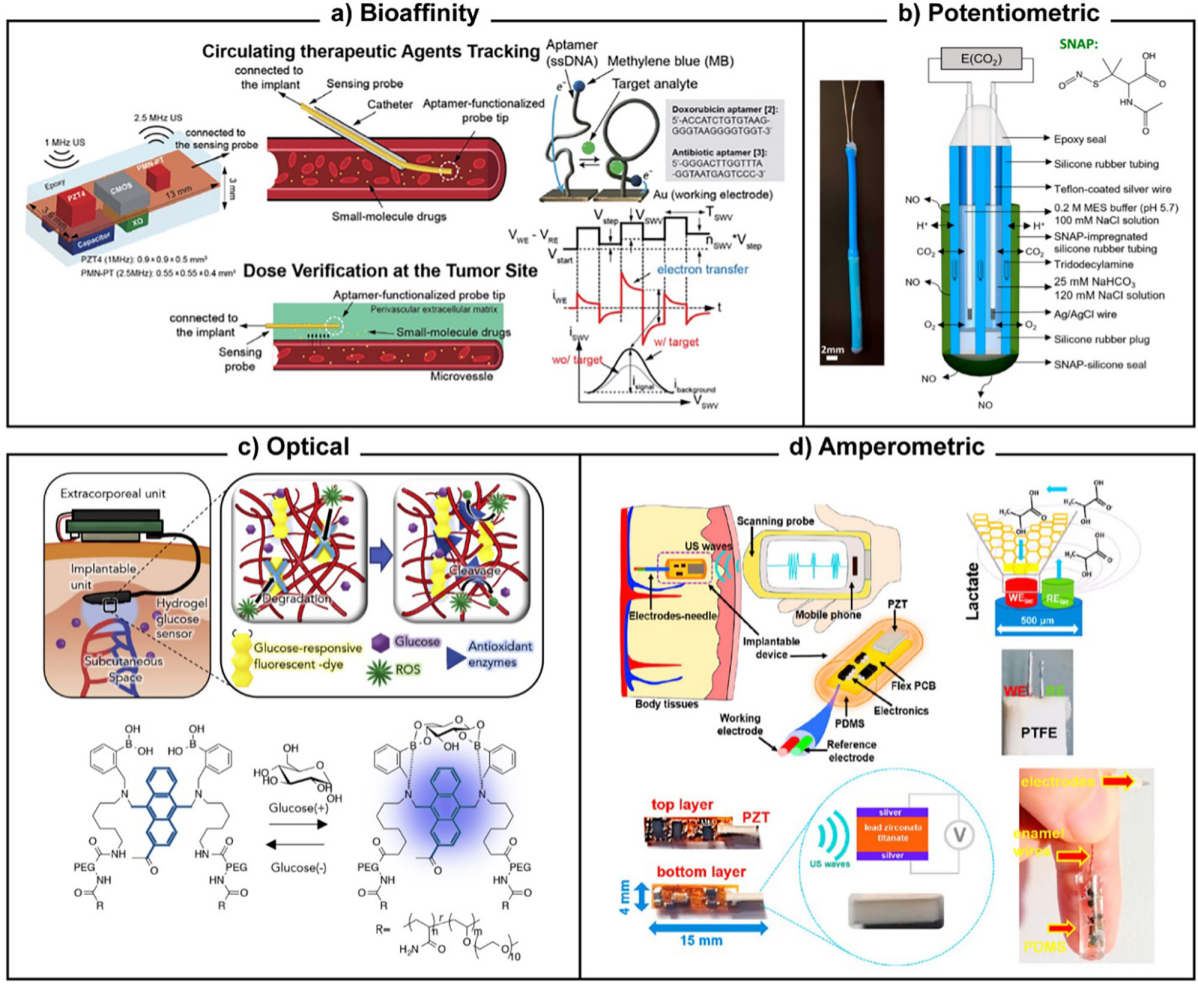
Implantable sensor examples for biochemical parameters. (a) Bioaffinity: a sensor probe for tracking therapeutic agents in the blood stream or verifying medication dose inside tissues depending on its biorecognition (aptamer) design. The figure illustrates both applications alongside the supporting sensor package as well as the biorecognition and measurement approaches. Reproduced with permission from Chien *et al.*, in *Digest of Technical Papers, Symposium on VLSI Circuits*, Kyoto, Japan (IEEE, 2019), pp. C312–C313. Copyright 2019 JASP.^82^ (b) Potentiometric: a sensor to monitor intravascular blood gas concentration, specifically CO_2_. The figure illustrates the sensing principle and packaging, focusing on its biocompatibility-enhancing drug release. Reproduced with permission from Zhang *et al.*, Anal. Chem. **92**, 13641–13646 (2020). Copyright 2020 ACS.^75^ (c) Optical: an interstitial glucose sensor leveraging the glucose concentration-dependent fluorescent properties of boronic acids. The figure illustrates the sensing concept, placement, and how integrated antioxidant enzymes reduce biofouling. Reproduced with permission from Sawayama *et al.*, iScience **23**, 101243 (2020). Copyright 2020 Authors, licensed under a Creative Commons (CC BY) license.^81^ (d) Amperometric: A sensor to monitor tissue lactate levels. The figure illustrates the operating principle, sensor construction, as well as data/power transmission. Reproduced with permission from Gil *et al.*, Biosens. Bioelectron. **182**, 113175 (2021). Copyright 2021 Elsevier.^79^

In Table III, we give an overview of the underlying working principles, as well as typical applications. In Figs. 3 and 4, we provide corresponding examples showing the breadth of the designs and approaches that characterize the field of implantable sensors. As shown, there may often be multiple different working principles to choose from for similar biomarkers or applications. Each has specific advantages in terms of performance or other engineering characteristics, which are discussed in detail in Sec. IV. The engineering requirements, in turn, are however entirely dependent on the clinical application. Subsection III B thus takes a closer look at the clinical categorization of implantable sensors.

**TABLE III.** Implantable sensor technologies: working principles and applications. This classification encompasses the most common types of implantable sensors, divided by type of biomarker (biophysical or biochemical) and the typical underlying sensing mechanisms.

Sensor type	Standard working principle	Applications	References
**Biophysical sensors**	Advantages: robust, simple, low power (except for electrophysiology and impedance). Disadvantages: highly sensitive to noise from other sources		
Temperature	Semiconductor with temperature-dependent resistance, or junction with gradient-dependent voltage	Predicting implantable device failure [Fig. 3(c)]; error correction for other sensors; monitoring local body temperature as an indicator of trauma or inflammation, or to guide hypo/hyperthermic treatment	54–56
Mechanical: motion	Inertia of miniature proof mass is transduced into electric signals in accelerometers or gyroscopes	Monitoring cardiac motion to assess global and regional function [Fig. 3(f)]; physical activity as a health measure or to tune stimulating implant (pacemaker, DBS) function	57–59
Mechanical: stress/strain	Ceramic generating deformation-dependent voltage (piezoelectric), or metal/semiconductor with deformation-dependent resistance	Blood pressure monitoring on the interior or exterior of a blood vessel [Figs. 3(b) and 3(d)]; bone or implant mechanical load monitoring; gastrointestinal motility; sound transduction for cochlear implants; some application interchangeability also with motion sensors	60–66
Mechanical: pressure/sound	Via strain (above), or using a cavity with a deformable enclosure and monitoring displacement using e.g., capacitance		
Electrical: electrophysiology	Electrodes recording electrical potentials generated by electroactive cells (cardiomyocytes, neurons)	Measuring neural function [Fig. 3(a)] to interface the brain with a computer or robot/prosthetic, or to tune stimulating implant function (DBS); cardiac function health monitoring or to tune stimulating implant (pacemaker, defibrillator)	9,67–69
Electrical/electrochemical: bioimpedance	Electrodes measuring electrical impedance of the surrounding liquid or tissue; partly biochemical due to the role played by ions	Differentiating cancerous from healthy tissue [Fig. 3(e)]; monitoring scar tissue formation around other implants to ensure function (stents, electrodes, etc.)	70–72
**Biochemical sensors**	Advantages: generally high specificity, good sensitivity. Disadvantages: reduced longevity, increased complexity		
Electrochemical: potentiometric	Electrode coated with ion-selective membrane, and potential measured against stable reference electrode, with the magnitude correlated to the selected ion concentration; alternatively, coating of the gate in a transistor geometry	Monitoring blood CO_2_ concentration as an indicator of respiratory failure [Fig. 4(b)]; pH to assess gastric health, tissue ischemia, or implant site inflammation/infection; detecting implant site inflammation and infection via pH changes; ion balance such as K^+^, Na^+^, and Ca^2+^ for broader homeostasis	73; 74, 75, 76
Electrochemical: amperometric	Under an applied potential, certain analytes can undergo electrochemical reactions at an electrode, with the current correlated to the concentration; in many cases, an enzyme is immobilized to first translate inactive analytes into redox-active species	Monitoring tissue lactate as a marker of patient health or athletic performance [Fig. 4(d)]; tissue oxygenation for similar purpose; glucose in diabetic patients	77–79
Photochemical: absorption, fluorescence, scattering	Target analyte interacts with an illumination source either directly, or via a photo-active reporter molecule	Continuous interstitial fluid glucose monitoring in diabetic patients [Fig. 4(c)]; blood and tissue oxygenation	80, 81
Chemical: affinity-based biosensing	Highly specific biorecognition element (cf. Sec. IV) specifically captures low-concentration target analyte, with readout typically optical or electrochemical (mechanical possible)	Therapeutic agent sensing for dose verification and tracking [Fig. 4(a)]; monitoring neurotransmitters to assess brain function.	82–84

### B. The clinical application of implantable sensors

**FIG. 5.**
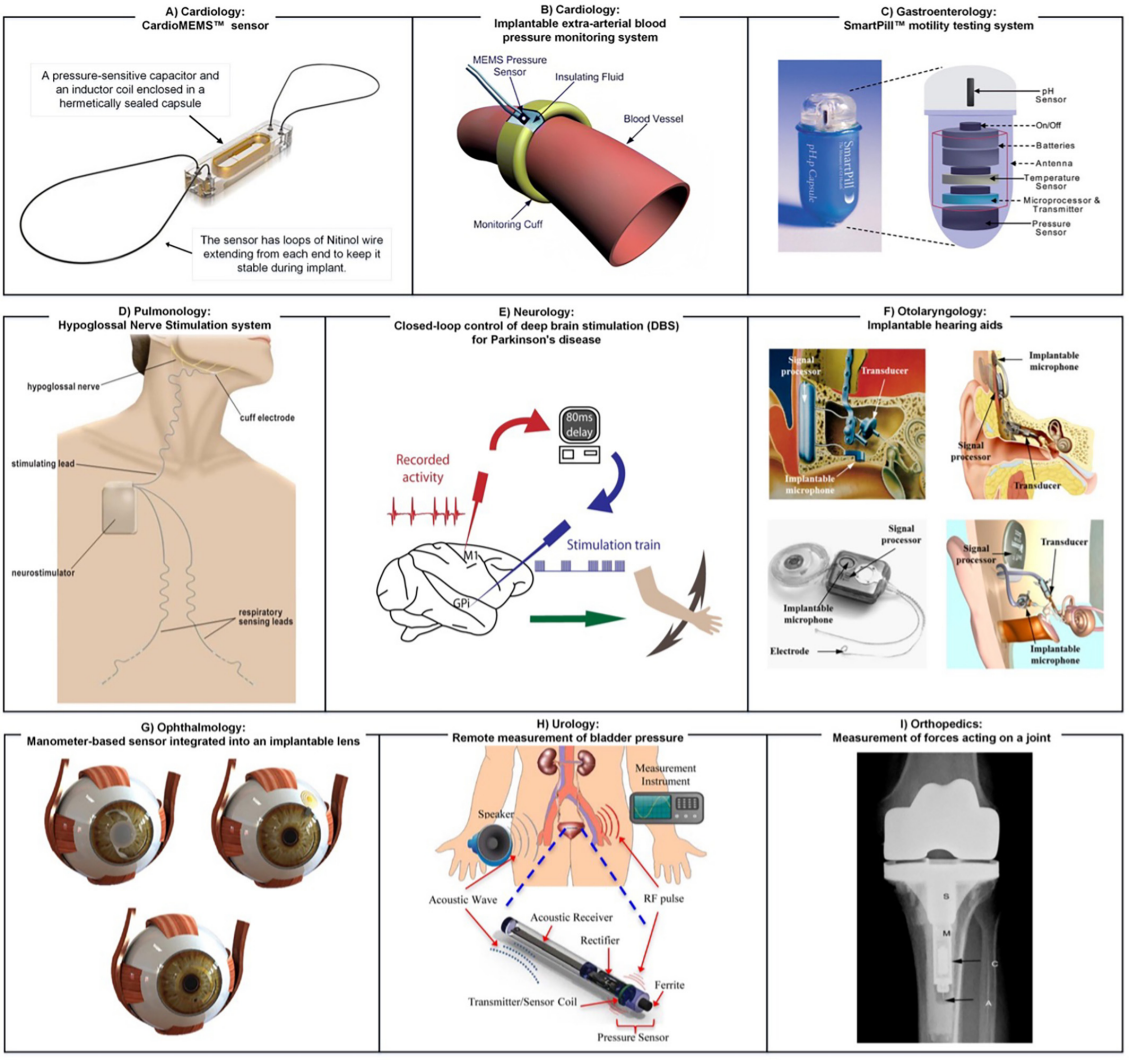
A visual representation of various applications of implanted sensors in different fields of medicine. (a) Cardiology: CardioMEMS™ sensor. Reproduced with permission Rali *et al.*, Am. J. Case Rep. **19**, 382–385 (2018). Copyright 2018 Authors, licensed under a Creative Commons (CC BY-NC-ND) license.^86^ (b) Cardiology: extra-arterial blood pressure (BP) measurement, a long-term arterial cuff developed at Case Western Reserve University. Reproduced with permission from Cong *et al.*, in *Proceedings of the 28th IEEE EMBS Annual International Conference, New York* (IEEE, 2006), pp. 1854–1857. Copyright 2006 IEEE.^87^ (c) Gastroenterology: wireless motility capsule that records pH, temperature, and pressure in real-time as it traverses the digestive system. Reproduced with permission from Fernandes *et al.*, Mater. Today. **12**, 14–20 (2009). Copyright 2009 Authors, licensed under a Creative Commons (CC BY-NC-ND) license.^88^ (d) Pulmonology: hypoglossal nerve stimulation system with sensing properties. Reproduced with permission from Eastwood *et al.*, Sleep **34**, 1479–1486 (2011). Copyright 2011 OUP.^89^(e) Neurology: a scheme of how a closed-loop deep brain stimulation (DBS) system with sensing properties is designed. Reproduced with permission from Santos *et al.* Neuron **72**, 197–198 (2011). Copyright 2011 Elsevier.^90^ (f) Otolaryngology: microphone technologies used for fully implantable hearing aids: top left: TICA (Implex, Munich, Germany); top right: Carina (Cochlear, Sydney, Australia); bottom left: TIKI (Cochlear, Sydney, Australia); bottom right: Esteem (Envoy, Saint Paul, MN, USA). Reproduced with permission from Woo *et al.*, Sensors **15**, 22798–22810 (2015). Copyright 2015 Authors, licensed under a Creative Commons (CC BY) license.^91^ (g) Ophthalmology: manometer-based sensor integrated into an implantable lens: top left: capacitive sensor measuring IOP directly; top right: resistive sensor on a contact lens; bottom: IOP, intraocular pressure. Reproduced with permission from Molaei *et al.*, J. Ophthalmic Vis. Res. **13**, 66 (2018). Copyright 2018 Authors, licensed under a Creative Commons (CC BY-NC-SA) license.^92^ (h) Urology: schematic of an acoustically powered transponder implanted in the bladder for remote measurement of bladder pressure. Reproduced with permission from Kim *et al.*, IEEE Trans. Biomed. Eng. **61**, 2209–2217 (2014). Copyright 2014 IEEE.^93^ (i) Orthopedics: postoperative radiograph of the knee joint demonstrating implantation of a second-generation instrumented tibial prosthesis that was used to measure forces acting on the joint. Reproduced with permission from D'Lima *et al.*, J. Biomech. **40**, S11–S17 (2007). Copyright 2007 Elsevier.^94^ The examples depicted in (g)–(i) are not directly discussed in this review. These examples were included in the figure to illustrate the diverse range of applications across various fields in medicine where implantable sensors are employed. The order of photos in this figure was intentionally arranged for visual impact rather than the textual order of mention.

Numerous types of implantable sensors can have a wide range of applications. The choice of the sensor is informed by the data required, the intended duration of use, the location of implantation, the invasiveness of the procedure, and patient characteristics such as age, weight, and health status. This section reviews clinically well-established application areas and the ways in which implanted sensors can provide valuable information and improve health outcomes. They operate by continuous monitoring of standard physiological parameters for informed decision-making by the doctor or patient [blood pressure, intracranial pressure (ICP), glucose, etc.], identifying biomarkers to facilitate early identification and hence the prevention of disease (cancer, infection, cardiovascular diseases, etc.), or by feeding sensor information back to other implant components that provide physiological stimulation (e.g., pacemaker, shunt, and insulin pump). Table IV provides an overview of implanted sensor use in different medical fields, with examples shown in Fig. 5. Note that these sensors also have risks such as potentially complex surgical procedures and associated complications, tissue damage, inflammation, and/or infection at the implant site, and failure of the device requiring repeat surgery.^85^

**TABLE IV.** Examples of implantable sensors in medicine: field-specific applications, advantages, and disadvantages. Table IV provides information on various types of implantable sensors used in different medical fields. It lists the advantages and functions of each sensor type, along with their possible benefits and applications. The references are also provided for further reading. As shown, implantable sensors have a wide range of applications in different medical fields, from monitoring heart activity to detecting cancer growth, and can significantly improve patient outcomes.

Field in medicine	Dominant sensor type(s)	Benefits and applications	References
Cardiology	Mechanical and electrophysiological cardiac monitors	Monitoring heart activity including heart rate variability and cardiac output, and detection of atrial fibrillation, arrhythmias, heart failure, and silent ischemia.	62, 86, 87, 95–103 [Figs. 5(a) and 5(b)]
Pulmonology	Pressure and gas sensors	Monitoring of upper airway patency, respiratory rate, and oxygen/carbon dioxide levels, and optimization of therapy delivery in patients with obstructive sleep apnea.	75, 89, 104–107 [Fig. 5(d)]
Neurology	Electrophysiological probes	Monitoring neural activity, detecting changes in brain function, measuring brain activity, neural signaling, electrocorticography (ECoG) signals, to guide stimulating implant function or prostheses.	90, 108–111 [Fig. 5(e)]
Oncology	Biochemical sensors	Monitoring of tumor response to chemotherapy and radiation therapy, detection of recurrent tumors, monitoring of tumor markers.	49, 82, 112
Diabetes	Amperometric and optical glucose sensors	Continuous glucose monitoring in patients with diabetes, including glucose variability and glucose prediction, providing information for insulin dosing.	113–115
Gastroenterology	Mechanical and biochemical sensors	Monitoring of gastrointestinal motility, food, or medication intake, detecting gastrointestinal bleeding or gastric and bacterial imbalance.	73, 88,116–122 [Fig. 5(c)]
Otolaryngology	Pressure (sound) sensors	Treatment of severe-to-profound sensorineural hearing loss, speech perception and understanding, sound localization.	91, 123–125 [Fig. 5(f)]
Ophthalmology	Pressure sensors	Long-term, direct monitoring of intraocular pressure changes for prediction and prevention of glaucoma and other ophthalmic diseases.	92, 126, 127 [Fig. 5(g)]
Urology	Pressure and motion sensors	Detecting small-scale autonomous movements (micromotions) in the bladder relevant to bladder physiology, potentially leading to improved diagnosis and treatment of urological conditions.	93, 128, 129 [Fig. 5(h)]
Orthopedics	Pressure and strain sensors	Monitoring of mechanical forces to track tendon healing after surgical repair, potential for improved diagnosis and personalized treatment of orthopedic conditions.	94, 130, 131 [Fig. 5(i)]

#### 1. Cardiology

Implantable sensors can monitor heart function and detect abnormal heart rhythms. For example, sensors can measure heart rate, cardiac output, and electrocardiogram (ECG) signals. Clinicians can use real-time hemodynamic data as an alternative approach to monitoring the status of heart failure (HF) patients and preventing potential hospitalizations.^132^ One example of a device marketed today is an implantable hemodynamic monitor (Chronicle, Medtronic Inc., Minneapolis, Minnesota)^100^ combining electrophysiological cardiac monitoring with a right ventricular pressure sensor. According to the COMPASS-HF trial, it led to a non-significant trend toward reduction in HF hospitalizations, emergency department visits, and urgent clinic visits. Other sensor insertion sites, such as the HeartPOD, a left atrial pressure sensor, that was shown to lead to a reduction in HF hospitalizations have been studied.^101,133^ However, HeartPOD's clinical trial was terminated before completion since the primary goal could not be reached, and there were too many implant-related problems associated with the transplantation technique.^101^ To date, the only available invasive biometric sensor that has also shown a statistically significant decrease in HF incidents is CardioMEMS (Abbott, Atlanta, GA, USA), a wireless implantable pulmonary artery pressure sensor [Fig. 5(a)]. This reduction in HF incidents has been reported in both clinical and real-world studies.^102,134^

Intrathoracic impedance assessments via cardiac implantable electronic devices (CIEDs) can measure a patient's heart rate (HR), HR variability, and even respiratory rate using electrophysiological and pressure sensing, making it valuable in the identification of arrhythmias and other conditions that could result in HF decompensation^102^ When used in conjunction with algorithms such as HeartLogic™, which uses scoring systems developed and validated from large multicenter datasets, CIEDs can identify HF patients at risk and, after further evaluation in more extensive clinical trials, may contribute to the optimization of HF management.^103^

According to the World Health Organization (WHO), hypertension affects roughly 30%–45% of the global adult population.^135^ Long-term blood pressure (BP) monitoring can be significantly improved by implantable blood pressure sensors that allow continuous BP tracking without interfering with daily activities.^62^ Continuous blood pressure monitors can be intra-arterial or extra-arterial.^97^ Current intra-arterial devices are small, wireless, catheter-based pressure sensors, which are bio- and hemo-compatible.^98^ However, the device placement inside the artery has been associated with a risk of clotting and dislocation. To address these issues, studies have suggested an extra-arterial blood pressure monitor [Fig. 5(b)] instead, which measures pressure indirectly through the arterial wall or through artery expansion and contraction.^63,97^ Several devices with various technological characteristics have been developed; however, the only device that has received US Food and Drug Administration (FDA) clearance for monitoring HF patients as of 2022 remains the intra-arterial CardioMEMS.^99^

Several uses of implantable BP monitors have been proposed, such as abdominal aortic aneurysm sac pressure measurement for surveillance after endovascular repair, which can help prevent leaks at the repaired site.^96^ In the field of coronary artery revascularization, implantable stent-based pressure sensors can be used as part of a “smart stent” system. When associated with a portable reader, this system can be configured to receive a signal detected by the implant during restenosis or stent thrombosis that warns the patient of the need for a complete diagnostic procedure and perhaps therapy.^64^

#### 2. Pulmonology and breathing

Implantable sensors are likely to revolutionize the way physicians monitor lung function and detect early signs of respiratory failure or disease. For example, sensors can be used to measure oxygen saturation and carbon dioxide levels.^104^ Current sensors can provide real-time monitoring of lung function and detect changes before they become critical.

The hypoglossal nerve, which controls the movement of the tongue, plays a critical role in maintaining the patency of the upper airway during sleep. Hypoglossal pacing, also known as hypoglossal nerve stimulation, involves the use of a device that delivers electrical stimulation to the hypoglossal nerve to enhance tongue movement and improve upper airway patency in patients with obstructive sleep apnea.^136,137^ The use of airway pressure sensors in conjunction with hypoglossal nerve stimulation allows for real-time monitoring of the effects of stimulation on upper airway patency by programming the implanted pulse generator connected to the electrodes placed on the hypoglossal nerve [Fig. 5(d)].^89^ This approach can be used to optimize the delivery of therapy. Hypoglossal nerve stimulation has been shown to be effective in reducing the severity of obstructive sleep apnea and improving sleep-related outcomes in patients who have not responded to other forms of treatment, such as continuous positive airway pressure (CPAP).^105,137^

Another promising field of interest is pulmonary arterial hypertension. Current clinical trials are investigating how data gathered by the CardioMEMS pressure sensor can be utilized for targeted rapid treprostinil (a prostacyclin analog that promotes vasodilation) therapy in patients with pulmonary arterial hypertension. It is believed that this approach may help improve right ventricular function and reverse right ventricular remodeling in participants with pulmonary arterial hypertension.^106,107^ In 2017, the FDA approved a fully implantable programmable intravascular delivery system for the parenteral administration of treprostinil. Since then, studies have shown that this implanted delivery device saves time, improves interpersonal interactions, and enables more independence, all of which are important parameters for patients.^138^ Ongoing trials testing targeted rapid therapy may encourage the development of a closed-loop intravascular delivery system that can achieve even better performance in these quality-of-life measures.

#### 3. Neurology and neurosurgery

Implantable sensors are becoming increasingly popular for monitoring neural activity and detecting changes in brain function. For example, sensors can be used to measure brain activity, neural signaling, and electrocorticography (ECoG) signals. These sensors are not only capable of detecting brain activity but can also be used to measure other physiological parameters such as temperature, pressure, and motion.^108^ One example of an implantable sensor is Neuralink's LinkV2, which is designed to record and stimulate neural activity in the brain. The company reported that this device can detect local neural activity, and when combined with spinal stimulation techniques,^139^ paralyzed patients who have suffered a spinal cord injury may in the future be able to operate certain devices,^140^ as was recently demonstrated by Lorach *et al.*^141^ Another example is the ECoG sensor, a type of implantable device used to measure the brain's regional and global electrophysiological activity. This type of sensor is often used in research and has been tested in clinical trials to detect changes in brain activity in patients with brain disorders such as epilepsy.^109^ In addition to these sensors, neural interfaces are also being developed that will allow bidirectional communication between the brain and external devices.^110,111^ It is possible that these interfaces could have a significant impact on the treatment of brain disorders, and perhaps enable the restoration of lost functions such as movement, sensation, and communication.

Deep brain stimulation (DBS) systems use electrodes surgically implanted in specific brain areas. There are approximately 150 000–200 000 people worldwide today with DBS implants.^142^ DBS systems are used to treat a variety of neurological conditions, including Parkinson's disease,^143^ essential tremor, dystonia,^144^ and obsessive-compulsive disorder (OCD).^145^ In particular, DBS of the subthalamic nucleus (STN) is a well-established form of therapy for Parkinson's disease.^146^ DBS of the globus pallidus interna (GPi) is used in cases of dystonia and for Parkinson's disease patients facing severe dystonia or dyskinesia.^147^ Currently, several closed-loop DBS systems are under development or in clinical trials. These systems can sense specific biomarkers such as movement intention (electrophysiology), tremor (accelerometer), and coherence between ECoG and accelerometer to trigger on-demand stimulation [Fig. 5(e)].^148,149^ One example is the “responsive neurostimulation” (RNS) system, which uses an intracranial electroencephalogram (iEEG) to monitor brain activity and adjust stimulation in real time.^150^ The FDA has approved the RNS system for the treatment of focal seizures.^151^

Vagal nerve stimulation (VNS) is a type of neurostimulation therapy that uses an implantable pulse generator to deliver electrical impulses to the vagus nerve.^152^ An implantable pulse generator is implanted under the skin of the chest and connected to the left vagus nerve through a small lead. The electrical impulses generated by the implantable pulse generator modulate the activity of the nerve and can cause changes in the function of the brain and other organs. VNS has been utilized to treat various neurological and psychiatric disorders such as epilepsy, depression, and anxiety.^153^ Some VNS devices, such as the AspireSR®^154–156^ operate as a closed-loop system that can automatically deliver stimulation in response to a sudden heart rate increase (picked up by electrophysiological sensors), which can serve as a predictor of an imminent tachycardia-based seizure. This system allows for automatic top-up VNS during seizures, which can help prevent seizures from spreading in the brain and reduce the severity and frequency of seizures in patients with drug-resistant epilepsy.

In addition to electrophysiological measurements, neurosurgeons and neurologists also aim to develop new types of shunts to treat hydrocephalus; i.e., excessive fluid accumulation in the brain.^157^ The most common type used in current clinical practice is the ventriculoperitoneal (VP) shunt, which is designed to drain excessive fluid to the abdominal cavity,^158^ thus reducing the ICP when it becomes too high. While standard shunts are based on mechanical resistance to pressure, such that when the pressure increases, a valve opens and reduces the ICP,^159^ in recent years, a new class of shunts have emerged in different stages of development, which integrate implantable sensors. These utilize electronic components^60,160^ such as flow and pressure sensors to maintain ICP at physiological levels^160^ and detect shunt failure.^161,162^

#### 4. Gastroenterology

Implantable sensors can be used to monitor gastrointestinal activity and detect changes in motility, pH, and nutrient absorption.^117^ Implantable sensors can also be utilized to detect conditions such as acid reflux or inflammatory bowel disease.^118^ One of the main advantages of implantable sensors is that they can provide continuous monitoring of the gastrointestinal system over an extended period of time.^119^ This allows for the collection of more accurate and detailed information than traditional diagnostic methods such as endoscopy or pH monitoring via a catheter.^120^ A prime example of a (temporarily) implanted sensor in the gastrointestinal (GI) tract is the wireless motility/pH capsule [WMC; Fig. 5(c)], an orally ingested FDA-approved device that continuously measures the temperature, pH, and pressure of its surroundings while traveling through the GI tract. In the future, it may become the method of choice for individuals suspected of having an abnormal gastrointestinal transit.^73^

Although still in the early stages of development, implantable food intake sensors are a novel technology that aims to replace traditional bariatric surgery for weight loss. These sensors can be placed in the stomach and track the amount of food consumed, sending data to a mobile app or another device for the patient and healthcare provider to monitor. When used in conjunction with a behavior modification program, these implanted sensors were shown to result in considerable weight reduction in obese individuals ^121^ and have helped people maintain their weight loss over time.^116,122^

#### 5. Hearing and otolaryngology

Implantable sensors have been developed to monitor inner ear function, detect hearing loss, and diagnose balance disorders.^123,124^ These sensors can be used to measure cochlear pressure, inner ear fluid levels, and acoustic signals. Cochlear implants, for example, are electronic medical devices that replace the functions of a damaged inner ear and help individuals with severe hearing loss to hear again.^163^ Cochlear implantation is a surgical procedure that involves the insertion of electrodes into the cochlea, coupled to a microphone (i.e., pressure sensor). While most systems on the market employ external microphones,^164,165^ fully implantable systems have recently become available [Fig. 5(f)].^91^ Clinical trials have demonstrated that these systems enhance speech comprehension and sound localization in individuals with severe to profound sensorineural hearing loss, and are safe and well-tolerated.^125,166^

## IV. BIOENGINEERING FUNDAMENTALS AND CHALLENGES OF IMPLANTABLE SENSORS

All types of physiological sensors, irrespective of whether they are implanted, wearable, or point-of-care, face challenges regarding their inherent clinical performance in accurately measuring biomarker levels. The types of implantable sensors presented above face additional and specific challenges with respect to biocompatibility, robustness, signal transmission, power sourcing, and more. In every case, however, they are almost entirely dependent on the specific application area. In the following sections, we elaborate on these challenges to provide the basic information and considerations for the design and fabrication of implanted sensors. In addition to the broad overview, we discuss the examples shown earlier in more detail. For an in-depth treatment of each topic, we refer the interested reader to the relevant topical reviews listed in the reference section.

### A. Sensor components

**FIG. 6.**
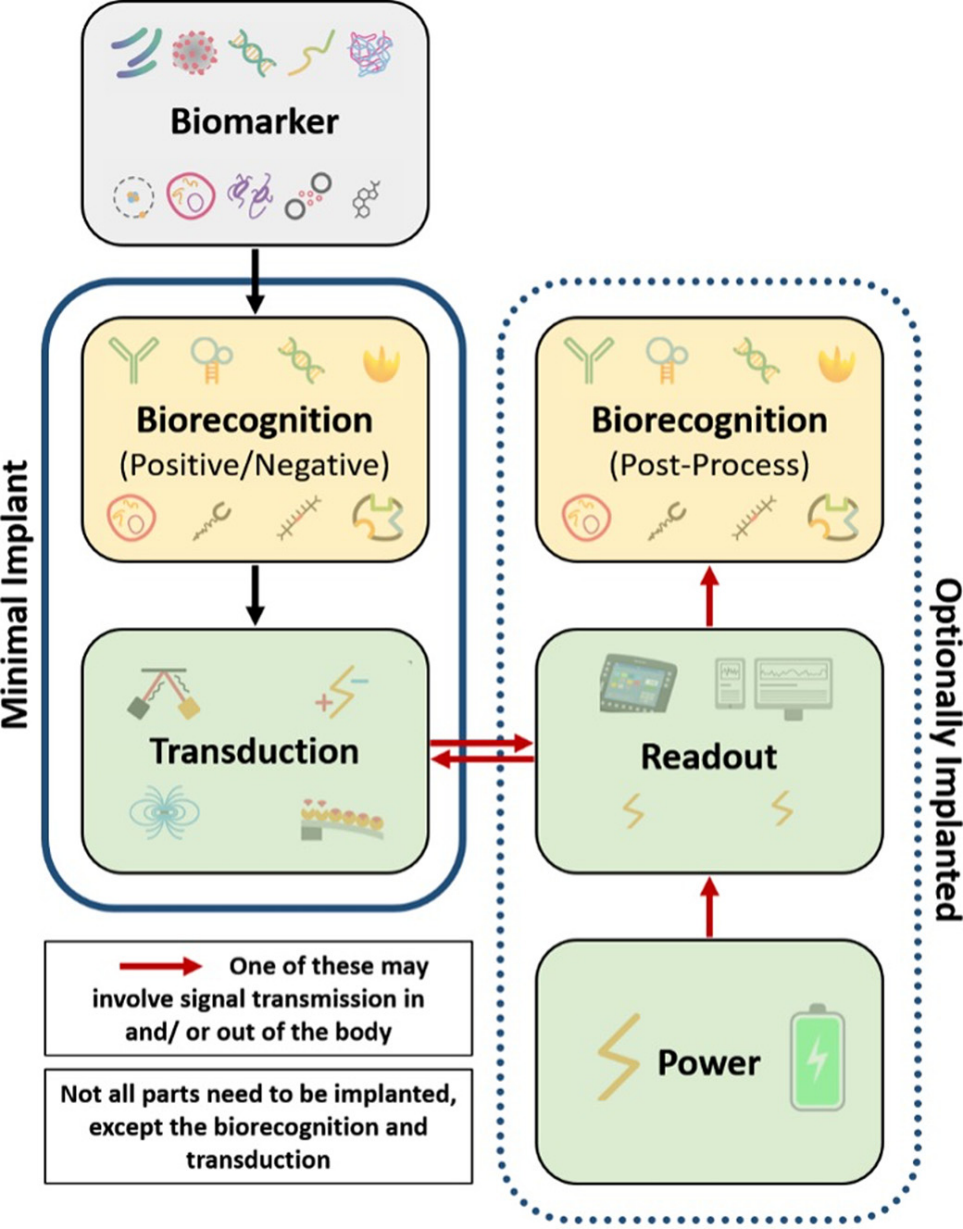
Conceptual diagram of an implanted sensor. Arrows indicate the general process flow, with one of the red arrows in many if not most cases having to pass from inside the body to the outside. A minimal implanted sensor will only contain the elements shown on the left inside the body; the optionally implanted elements are indicated on the right. Specific biorecognition of the biomarker can take place as on-sensor (positive or negative) or as off-sensor post-processing; at least one is required, though often both will be present in some form. See the text for additional details.

To better grasp the engineering challenges inherent to implantable sensors, a brief introduction to the conceptual sensor elements and how they interact is in order. The core function of a sensor is to turn changes in a biomarker into a measurable signal; e.g., changes in blood pressure or glucose into changes in voltage or current.^167^ This is termed transduction. For a sensor to be useful, this transduction needs to be made specific to the biomarker, which is the domain of biorecognition. Together, they constitute the working principles discussed in Table III. Biorecognition can be further divided into two broad classes: positive/negative biorecognition relying on “real” biochemical elements placed on the transducer, and post-process biorecognition relying on a “virtual” analysis of the transducer signal. In either case, readout instrumentation is required to provide transduction input energy (e.g., electrical potential or optical illumination) and to record the signal. These processes naturally require a power source or generator. These conceptual elements and relations are illustrated in Fig. 6.

One key feature of implantable sensors, as captured in the schematic, is that not all the conceptual components need to be part of the same unit. Only transduction needs to be in direct contact with the biomarker inside the body (as does positive/negative biorecognition, if present). For purely sensing applications, only the data about biomarker levels needs to be available to patients and/or caregivers outside the body. All the other components can then be placed on either side of the internal/external divide, with at least one pathway crossing it (red arrows). Conversely, when sensors feed back into the stimulating implants (pacemaker, DBS), even the data can remain inside the body. These constraints, and possible design choices, give rise to many of the unique engineering challenges complexifying the design of implantable sensors on top of those found in all wearable or point-of-care sensors; challenges that we will discuss in more detail in Secs. IV B–IV H.

### B. Sensor performance, biorecognition, and transduction

The ultimate measures of implanted sensor performance are real-world studies in broad patient (and control) populations, where the sensor is compared against current gold-standard assays for the biomarker, and where the impact of sensor data on clinical decision-making and outcomes is evaluated. Reaching this point in implantable sensor development is a lengthy and expensive process, and one of the adoption challenges (cf. Sec. V). In the initial conceptualization and testing periods, simpler analytical measures are preferred and can function as imperfect predictors of such clinical performance. Herein, we will discuss (analytical) sensitivity and selectivity: broadly, that the sensor produces a maximal signal in response to the targeted biomarker and a minimal response to other stimuli, respectively.^168^

Selectivity largely depends on biorecognition. In the case of biophysical biomarker*s* (Table III; Fig. 3), the function of the transducer is simply to convert from one physical domain into another more easily digitized one (typically electrical; see sensitivity). This means, for instance, transforming changes in implant temperature into electrical resistance [Fig. 3(c)], or mechanical fluid pressure into changes in electrical capacitance [Fig. 3(d)] or voltage [Fig. 3(b)]. In the case of electrical biomarkers [Figs. 3(a) and 3(e)], the transducer may simply function as an amplifier. Biorecognition can then rely largely on “post-process” analysis of the transducer signal to filter out undesired interference or noise from the biomarker response. This approach requires robust knowledge of the physiological parameter space of biomarker levels over time (frequency, amplitude, and etc.), and similar insights into the characteristics of possible interferents.^169,170^ The clearest examples come from electrophysiological data [Fig. 3(a)], where the particular characteristics of neural spikes make it possible to differentiate them from unspecific background noise and drift. Since it is a purely virtual approach, it has immense advantages: (1) It greatly simplifies sensor architecture compared to on-sensor biorecognition (see next paragraph), reducing associated engineering challenges across the board. This is one of the primary reasons why current implanted sensor applications are predominantly in the biophysical parameter space (cf. Sec. III). It can also work in tandem with on-sensor biorecognition to improve selectivity for biochemical biomarkers.^171^ (2) Software algorithms can be innovated and refined much more cheaply and rapidly than changes in sensor hardware. Advances in deep learning, neural networks, and artificial intelligence all provide vast opportunities for further sensor innovation.^172,173^ The NeuralTree classifier for brain electrophysiological data, for example, has proved highly successful in detecting a wide range of events-of-interest, including epileptic seizures and Parkinsonian tremors.^174^ Its applicability is unconstrained by the underlying sensor design, and—with feedback-control of DBS in mind—it is designed for functionality even in miniaturized processing electronics. Alternatively, algorithmic development can also enable new functionalities from existing sensors, for example, one study employed a (wearable) continuous glucose sensor as a diagnostic tool to classify study participants as diabetic, healthy, or pre-diabetic, with an area-under-curve of 0.86.^175^ The greatest challenge facing this type of biorecognition lies in obtaining a sufficient understanding of the expected signal and interference signal characteristics, so as not to misclassify signals in either direction. Secondary sensors, which are geared toward explicitly measuring potential interferents, are another useful strategy here,^176^ at the obvious cost of added complexity.

In the case of biochemical biomarkers (Table III; Fig. 4), what we term “positive” biorecognition, i.e., the use of a coating on the transducer surface that selectively binds or interacts with the biomarker, remains the norm.^177^ Very high levels of selectivity can be achieved using “lock-and-key” macromolecules (i.e., affinity-based sensors). These can be biologically evolved, such as antibodies or nucleic acids, or analogous bioengineered alternatives such as aptamers [Fig. 4(a)] or molecularly imprinted polymers. *Ex vivo*, such approaches facilitate, for example, the detection of sub-femtomolar protein concentrations even in complex fluids like blood plasma.^178^ One notable downside of strong biomarker capture is poor reversibility, and hence poor dynamic performance (required for implant use) unless additional measures are taken.^83^ The other types of biochemical sensors listed in Table III rely on more transient interactions facilitated by biorecognition elements ranging from biological [enzymatic reactions, such as lactate oxidase in Fig. 4(d)] to physicochemical [e.g., molecular complexing, such as boronic acid-glucose in Fig. 4(c), or ionic membrane interactions in Fig. 4(b)]. For any type of positive biorecognition, the key challenges are the stability and longevity of the biorecognition element itself and its tethering to the transducer surface,^179^ particularly given the complex environment around an implant. As discussed in the sections on biocompatibility and lifetime, this is a prime concern for the entire sensor implant. However, molecular-scale biorecognition structures, which are often based on biological or biochemical building blocks, are much more sensitive to these influences than typically larger-scale and non-biological transducers. These concerns have also driven research into sensors for biochemical biomarkers that rely on the differential response of multiple non- or poorly selective sensors (which may be more robust) coupled with post-process biorecognition,^180,181^ an area of research often termed chemometrics or the electronic tongue.

Finally, both biophysical and biochemical biomarker recognition can be enhanced by shielding the sensor from interferents (negative biorecognition). This can include physical shielding from vibrations or electrical noise [such as the electromagnetic shielding tube in Fig. 3(c)].^182^ It can also involve semi-selective membranes to exclude certain biochemicals from the sensor surface based on charge or size [e.g., silicone tubing in Fig. 4(b) allowing only gas/ion diffusion].^183^

Sensitivity is by definition linked more closely to the transducer, being the unit transducer output change (e.g., capacitance, voltage, wavelength, and intensity) per unit biomarker change. For practical purpose, a slightly broader interpretation that also covers how accurately the transduced signal can be read out is useful to be able to compare different types of transducers. Arguably, however, sensitivity is not the prime hurdle to implantable sensor development. Most biomarker targets that are sufficiently widely applicable to warrant an implant (see, e.g., Table III) exhibit clinically relevant changes that far exceed the sensitivity available from optimized sensor architectures. For instance, glucose sensor sensitivity has been reported in excess of 10 mA/mM/cm^2^,^184^ whereas clinically actionable changes are >0.1 mM (i.e., >0.1 mA for an implant-suitable 10 mm^2^) and readout instrumentation can easily achieve sub-*μ*A accuracy. Similarly, a pressure sensor sensitivity of ∼500%/mm Hg has been reported,^185^ compared to accurate readouts for <10% resistive or capacitive changes and actionable blood pressure changes of >1 mmHg. By contrast, selectivity and biocompatibility impose greater limitations. For a given transducer type, a larger size generally increases sensitivity,^186^ but also increases the biocompatibility concerns (see below). Between transducer types, electronic outputs can generally be read out more accurately and with smaller readout instrumentation than optical, magnetic, or other outputs [compare Figs. 3(c), 4(a), 4(d) to 4(c)]. For implantable sensors with on-sensor readout, it is thus unsurprising that electronic-parameter-output transducers continue to dominate the market. This also applies to off-sensor readout, albeit for different reasons (see sections on power and data transmission).

Transducer choice is also impacted by the type of biomarker. For mechanical biomarkers like pressure or force, largely equivalent optomechanical and electromechanical transducers can be designed where the major difference lies in the readout. Similar principles also apply to most bio-affinity sensors. By contrast, electrical biomarkers (potential, impedance) inherently lend themselves to electronic transduction, as do electrochemically active biomolecules (or where relevant enzymes are available). Conversely, optical transduction is ideal for molecules with strong optical interactions (or where relevant photochemical indicators are available). In the case of glucose or oxygen, for example, both routes are available. Electrochemistry may, however, become a biocompatibility concern in implantable sensors because non-target molecular reactions at both the working and counter electrodes are poorly controlled. Given the recent advances in optical component miniaturization,^80^ such concerns may favor optical readout.

### C. Biocompatibility and biofouling

These two terms, though imprecise, describe two aspects of host-implant interactions: biocompatibility concerns the extent of adverse effects (or the lack thereof) that an implanted sensor may have on biological functions, and biofouling refers to the adverse effects of physiology on sensor function. Both types of adverse effects are unavoidable; proper sensor engineering and placement can however attempt to minimize them.

An ideal implant (as well as its surgical implantation procedure) should obviously aim to produce minimal damage to the surrounding tissue and avoid direct interference with physiological processes (e.g., minimal obstruction of a blood vessel if placed inside one). These straightforward considerations make it clear that implant size is a critical factor. Other biological interactions are more difficult to account for. After implant placement, in less than minutes, proteins from the surrounding fluids begin covering the surface, followed by an inflammatory “foreign body” cell response over the course of hours to weeks.^187,188^ The specifics depend on the implant location, with the brain and its immunoprivileged status the most notable special case.^189,190^ On a timescale of weeks to months, the inflammatory response will then decline as the implant is compartmentalized away from physiological processes by means of a fibrotic capsule. Both biocompatibility and biofouling are intertwined with these processes (as are the strategies for alleviating them), though they are not identical.^191^ For biocompatibility, the biggest concern has to do with the inflammatory response, since this can propagate adverse effects throughout the body. Conversely, for biofouling, the locally occurring protein and cell adhesion, as well as eventual fibrous encapsulation present the greatest challenges: they can dampen the mechanical response, reduce chemical diffusion, and shield electrical potentials, thus overall degrading the sensitivity.

One factor in the host body response is bulk stiffness. A mismatch in stiffness between the sensor and the adjacent tissue can strongly contribute to an adverse host response.^192^ While the 100+ GPa stiffness of standard inorganic sensor construction materials (semiconductors, metals) may be a reasonable match for bone [or its replacement as in Fig. 3(c)], it is ill-suited for many other body tissues, which range from low kPa [brain; Fig. 3(a)] to MPa [blood vessel walls; Figs. 3(b) and 3(d)].^193^ This has driven considerable research into the construction of soft and even shape-adaptable sensors, or transducers, which are also of great interest in the wearable space.^194^ A recent example is the NeuroString, an amperometric neurotransmitter sensor.^195^ Based on elastomer-embedded graphene (sub-MPa), it exhibited significantly lower colonic disruption compared to a similar-sized polyimide (∼GPa) probe in rats. Even though stiffness is a bulk property, employing soft outer packaging around solid-state inner components, may in itself improve mechanical compatibility with soft tissues [silicone or PDMS in Figs. 4(b) and 4(d)]. Due to the difficulties associated with all-flexible power, readout, and/or transmission electronics, this remains a common strategy when such components are part of the implant.

Topography, surface energy/charge, and biochemistry are the other main considerations for biocompatibility/fouling and are purely surface-related. It is thus significantly easier to modify them with various coatings. Nanometer-scale smoothness can beneficially modulate surface–protein interactions (and thus initial inflammatory response), whereas 1–5 *μ*m-scale structures may reduce cell adhesion and thereby mitigate longer-term effects.^192,196^ Akin to nanometer-scale topography, surface energy and charge are additional key factors in modulating protein interactions, with hydrophilic surfaces generally being more biocompatible. Passive or inert biocompatible/low-fouling materials and coatings exploit these types of correlations. Options range from surface-structured titanium to simple coatings made of parylene or silicone to polymer-modified surfaces. Polyethylene glycol (PEG) in particular has been a long-term staple on the latter front, but, more recently, it has raised concerns with respect to degradation and immunogenicity under certain conditions. Recent research has yielded highly promising results with zwitterionic polymers and hydrogels, as well as with modified alginates.^188,197^ Jayakumar *et al.*, for instance, developed a zwitterionic coating that outperformed common glucose sensor coatings (including PEG) in terms of protein adsorption and cell adhesion.^198^ It also featured up to an order of magnitude lower adverse impact on glucose sensitivity. This also highlights the typical trade-off impacting development: between improving biocompatibility, biofouling, and broader homeostasis on the one hand, and the inevitable degradation of analytical sensor performance (from a slowdown in diffusion in this example, but also mechanical dampening, optical absorption, etc.) on the other.

Biochemical surface modifications are a strategy that takes polymeric modifications even further toward more actively modulating the host response by employing biological signaling cues. These materials do not attempt to remain fully separate from the surrounding physiological environment, but rather integrate beneficially with it.^199^ This may entail mimicking the extracellular matrix of the surrounding tissue^192^ or the inclusion of drugs or growth factors that suppress the inflammatory response.^191^ The potentiometric blood vessel CO_2_ probe shown in Fig. 4(b), for example, generates nitric oxide to prevent platelet adhesion and thrombus formation. Conversely, the optical glucose sensor shown in Fig. 4(c), incorporates antioxidant enzymes, that protect the chemical biorecognition element from degradation. Even more so than with polymeric materials, however, devices employing bioactive materials present difficulties regarding sterilization.^200,201^ Finally, there have been efforts to actively modulate biofouling. These aim to displace adsorbed proteins and cells by employing mechanically actuated shear, reversible state-change materials, and similar actuation.^191,202^ While not employed on sensors in clinical use to date, this presents intriguing possibilities for future advances.

### D. Implantation and localization

The placement of an implantable sensor factors into all the considerations discussed in this section. The optimal location needs to consider tradeoffs in sensitivity (the best access to the biomarker), selectivity (the lowest interference), biocompatibility (the least host response), biofouling (the least sensor degradation), and power/data transmission (the highest efficiency). Three additional considerations need to be discussed in more detail.

The first is the relative operative ease of placing the implant at the target location. The size of the implant plays a key role here since a smaller sensor can be implanted with less invasive surgery in a wider range of locations. In ascending order of invasiveness, the options range from needle/probe injection (most feasible for placement close to external body surfaces, such as the Eversense optical glucose monitor),^114,203^ catheter placement (e.g., CardioMEMS and most other cardiac and intra-blood-vessel sensors),^106^ to endoscopic and open surgery.

The second consideration is that a traditional implantable sensor is designed to optimally perform in one given location over time; it may also need to be retrieved at the end of its lifetime (see Sec. IV F). Thus, it needs to be prevented from inadvertently migrating in the liquid and soft environment of the body due to the highly dynamic movement throughout the day. Fixation with stiff metal anchors in the surrounding tissue presents clear biocompatibility concerns in most cases, with the natural exceptions of intrinsically stiff bone or inside blood vessels [Fig. 3(d)].^204^ Sutures [applied, e.g., in Fig. 3(f)] are more mechanically compliant but cause additional tissue strain and disruption. Sensors aimed at tubular targets (nerve bundles, blood vessels) can also be designed as cuffs that wrap around the outside of that biological structure [Fig. 3(b)].^63^ For many applications, however, biocompatible glues and adhesives present the best option.^205,206^

The third concern relates to certain sensors that are intentionally designed to traverse the body [i.e., ingestible capsules; Fig. 3(e)]. For these sensors, the challenge shifts from fixation to tracking to enable the physician to correlate sensor data with physiological location.^207,208^ In principle, tracking can be considered a more demanding application of power and information transmission, which is discussed in Secs. IV E and IV F.

### E. Data transmission

Regardless of how sensor components are implemented, the information regarding the biomarker inside the body will often need to be interpreted externally (except when signals are used in conjunction with closed-loop control of a stimulating implant such as a pacemaker, DBS, etc.). A trivial solution is employed in tethered sensors, where the physical sensor package extends from the inside to the outside of the body [using wires or optical fibers, as seen in Fig. 3(b) or Fig. 4(c)].^209–211^ This allows for a simple exchange of electrical or optical signals across bodily barriers, with the major disadvantage of permanently disrupting them, thereby creating new biocompatibility issues (including potential infections). Wireless systems are thus the obvious preference.

One wireless possibility is to only implant a minimal sensor (transducer, biorecognition if relevant) and employ external readout. This relaxes the engineering constraints for instrumentation (and power) considerably in terms of size and biocompatibility since they transition from implanted to wearable. The most common approach is inductive–capacitive (LC) resonators.^212,213^ In this case, biomarker recognition—such as pressure in Fig. 3(c)—is transduced into a shift in capacitance, which in turn alters the resonant frequency of the LC circuit and can be interrogated using an external antenna. However, only a small number of biomarkers/sensors are suitable for such minimal systems. More applications become accessible with systems that employ minimal on-sensor transduction/readout circuitry (instead of a direct resonant transducer) to modulate the interrogation signal.^212,213^ This generally takes the form of a passive RFID (radio frequency identification) chip operating in the *μ*W power range. Analogous magnetic [Fig. 3(c), where the metal implant prevents the use of electromagnetic waves] or ultrasonic implementations [as seen in Fig. 4(b), where lactate concentration ultimately correlates with a backscatter phase shift] are also possible when deep-tissue or higher-power applications are required.^214,215^

**FIG. 7.**
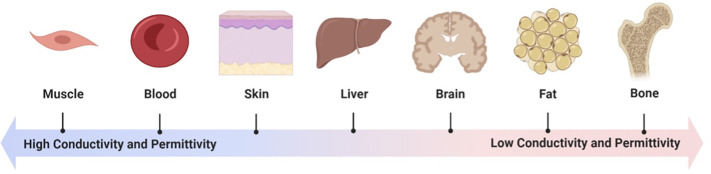
Dielectric properties of various tissues at 2.45 GHz (based on Table V). Figure created with BioRender.com.

Most implanted sensors employ more actively powered (cf. Sec. IV F) instrumentation instead to enable a broader range of readout. With reduced power constraints, technical advances in communication chip design can be leveraged using off-the-shelf components and standardized protocols such as Bluetooth Low Energy and ZigBee.^212,213^ Their two shortcomings are their mW-level power consumption and their generalized (rather than implant-optimized) nature. For implants relatively close to the skin, infrared optical signal transmission has also shown promise.^216,217^ Alternatively, custom radio frequency transmission protocol and antenna development can be pursued. A key consideration here is the propagation of electromagnetic signals through the human body. Attenuation is dependent on the dielectric properties of the tissues, including conductivity (higher is worse), permittivity (lower is worse), and thickness (higher is worse), as well as deflection, which is dependent on interfaces between the layers.^218^ As shown in Table V and Fig. 7, these can vary widely with tissue type and signal frequency^219–221^ and hence require careful consideration.

**TABLE V.** Comparison of dielectric properties across various tissues at 13.56 MHz and 2.45 GHz. ε_r_ = Relative permittivity, σ =  conductivity (S/m). The selected frequencies are some of the most commonly utilized in data transmission: 13.56 MHz is used for NFC/RFID, with low power and short range, and 2.45 GHz is used for Bluetooth/ZigBee/WiFi, with higher power and longer range for faster data transmission. The table shows that different tissues have different electrical properties, which can impact the behavior of electromagnetic fields in these tissues. The data in this table are based on information reproduced with permission from Pethig *et al.,* IEEE Trans. Electr. Insul. **19**, 453–474 (1984). Copyright 1984 IEEE.^222^ See Fig. 6 for the corresponding illustration.

Tissue	13.56 MHz (NFC/RFID)		2.45 GHz (Bluetooth/ZigBee/WiFi)	
	**ε_r_**	**σ**	**ε_r_**	**σ**
Blood	155	1.16	60	2.04
Bone	11	0.03	4.8	0.21
Brain (white matter)	182	0.27	35.5	1.04
Brain (gray matter)	310	0.4	43	1.43
Fat	38	0.21	12	0.82
Liver	288	0.49	44	1.79
Muscle	152	0.74	49.6	2.56
Skin	120	0.25	44	1.85

### F. Power sources

As mentioned above, more power generally equates with a wider range of possible transduction and readout modalities. These fall into three main categories: batteries, generators, and wireless.

Battery technology is making constant progress, but as is evident in mobile phones and electric cars, their miniaturization lags drastically behind circuits and sensors. Single-use batteries are the simplest, with today's lithium-based cells currently providing 10+ years of use for *μ*W-level cardiac implants (nuclear batteries can provide even longer lifetimes, albeit at lower power).^223^ For continuous sensing and transmission, however, higher long-term power may be required, which calls for rechargeable batteries or supercapacitors.^224,225^ The requisite recharging is then handled wirelessly.

A natural alternative to batteries is harnessing available mechanical, chemical, or thermal energy inside the body.^225^ Power in the *μ*W range can be generated from body- or organ-level motion using mechanical-to-electromagnetic coupling; for example, from the blood flow using miniaturized turbines, or from local repetitive compression or shearing using piezoelectric or triboelectric generators.^223,226^ The latter currently dominates implantable applications as far as energy harvesting is concerned, though translation into clinical practice is still not common. The blood pressure cuff shown in Fig. 3(b) is one illustrative academic example, where the piezoelectric transducer can simultaneously function as a power harvester. This sensor-generated energy can even be fed directly back into a stimulating implant, as demonstrated by Shlomy *et al.* for linking a triboelectric mechanical sensor with neural stimulation to restore tactile sensation.^10^ Biofuel cells employ electrode-coupled enzyme electrochemistry to generate similar power levels but currently suffer from poor long-term stability. Thermal gradients can be exploited using thermocouples without fragile or moving parts but are often unavailable at the implant site.

Finally, wireless power transfer can be employed either continuously for direct sensor operation or intermittently to re-charge an on-sensor battery or supercapacitor. Inductive near-field coupling is currently the dominant mechanism in this field since it can deliver mW-level power over cm-scale distances into the body.^213,225^ As with information transmission, the tissue type plays a large role in any radio frequency transfer efficiency (cf. Table V). Capacitive near-field and optical power transfer may be able to achieve higher power transfer efficiencies and/or have advantages in terms of the antenna (and thus implant) size but are limited to applications close to the skin. For deeper implants, the potential options include radiative (mid-field) electromagnetic waves, ultrasound [Fig. 4(a), which is a deep-tissue application], or magnetic fields [Fig. 3(c), where the metal implant prevents the use of electromagnetic waves], but these are generally still in their infancy.

### G. Lifetime and degradability

The final consideration is the implant lifetime. From a design point of view, the ideal goals for an implantable sensor vary widely, depending on the application, and range roughly from weeks to a human lifetime. Achieving truly life-long implants is currently impeded by the deterioration of sensor packaging and components inside the challenging body environment, and that of batteries over time (whether single-use or rechargeable). Considering cardiac pacemakers as an exemplary, extensively studied implant (incorporating electrophysiological sensing), one large study reported ∼20% failure rate at 6 years, 20% of which was attributed to non-battery failure.^227^ Sensors for biochemical parameters suffer from lifetimes shorter by a factor of 10, with the optical Eversense glucose monitoring system setting a 180-day clinical practice benchmark.^114,115^ This gap is in large part due to the fragility of on-sensor positive biorecognition. Possibly, the longest-functioning biochemical sensor was described by Gough *et al.*, measuring glucose amperometrically up to 1.5 years (n = 1, pig).^228^ Their architecture implements several anti-biofouling features (and, due to power requirements, a battery) in a package that is 80 times larger than the Eversense. Thus, the difference in patient burden from repeat implantations vs biophysical sensors remains significant, and it will require further engineering advances to alleviate this issue.

For shorter-term applications, the added patient burden does not arise so much from the need for replacement but for terminal removal. This can be addressed by designing the entire implant to undergo controlled biodegradation by the body; i.e., by taking active advantage of the challenging body environment and its normally undesirable foreign body response.^229^ Degradable design clearly places much more stringent requirements on all the materials involved, beyond (mainly) the outer surface as is the case for standard biocompatibility. Rather, each material—and its degradation products—needs to be biocompatible, with degradation rates engineered to match the desired sensor lifespan. Organic polymers (both natural and synthetic) fortunately present a large design space that can be taken advantage of.^230^ Electronic components can use conjugated polymers,^231^ or some of the essential biological metals (magnesium, molybdenum, zinc, etc.). Certain semiconductors can also be implemented in the form of thin layers. One of the most impressive examples to date is the multi-sensor brain probe by Yang *et al.*^232^ It is constructed from silicon, silicon oxide, magnesium, molybdenum, and tungsten or molybdenum sulfide on a polymer substrate, all fully degradable within 1–2 months. For the first 3–4 weeks, this probe can measure temperature, electrophysiology, pH, and amperometric dopamine.

### H. Are these engineering challenges insurmountable?

In summary, implantable sensors present a multitude of engineering challenges. It is worth emphasizing that none of the individual challenges are unique to this particular field or application area, as evidenced by the works cited throughout this section. Research on biocompatibility is advancing rapidly, propelled by medical implants in general. Transducer and power source miniaturization are driven by sensors ranging from the field of food safety to the internet-of-things. Implantable sensors, however, face a uniquely broad combination of challenges. Their real-life application thus somewhat naturally lags behind passive implants and portable/wearable sensors. Nevertheless, the developments in these adjacent fields hold the promise that the engineering challenges can ultimately be overcome and that other factors related to clinical and patient considerations will prove the main deciding factors in their wider adoption.

## V. EXPLORING THE GAP: EXAMINING THE DISCREPANCY BETWEEN IMPLANTABLE SENSOR RESEARCH AND CLINICAL ADOPTION

**FIG. 8.**
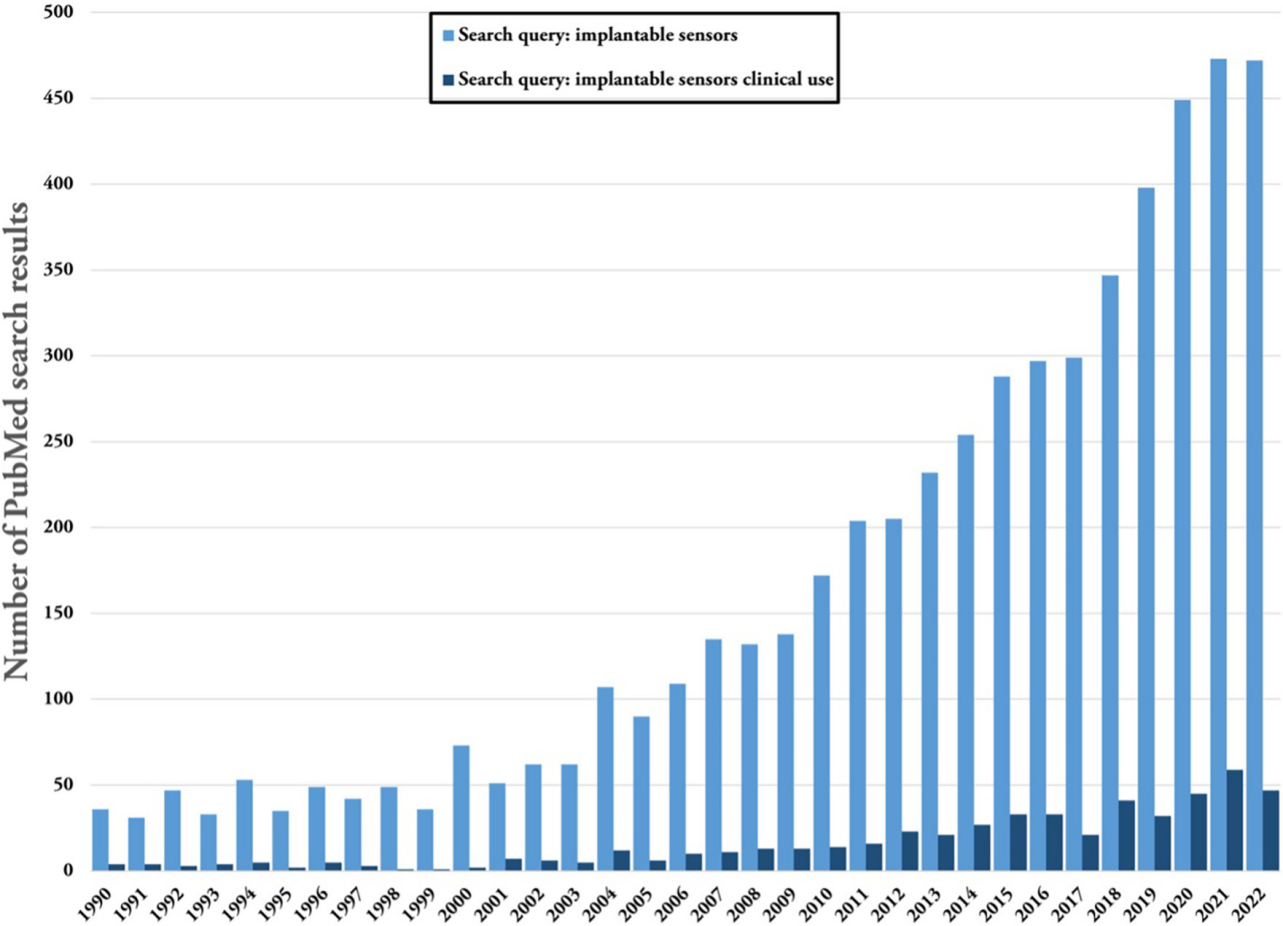
Graphical representation of the PubMed search results on implanted sensors. The graph shows a significant difference in the number of studies conducted on implanted sensors compared to the number of studies on their actual use in clinical settings. To ensure comprehensive coverage of the available literature, the search query employed various synonymous terms for “clinical use,” including medical application, therapeutic use, patient treatment, and related terms.

As shown in Sec. III, implantable sensors can improve patient care significantly by continuously monitoring vital signs and disease development. However, as shown in Fig. 8, there is an enormous disparity between the number of published articles in English dealing with these devices and their actual use in clinical settings. This disparity becomes much more pronounced when the number of FDA approvals for implanted sensors is factored in. A comprehensive search of the FDA database^233^ was conducted using 15 different terms associated with implantable sensors; namely, “implanted sensor,” “implantable sensor,” “implant sensor,” “body-implanted sensor,” “swallowable sensor,” “biosensing implant,” “embedded biosensor,” “subcutaneous sensor,” “intravascular sensor,” “intracranial sensor,” “implantable telemetry device,” “implantable monitoring device,” “internal sensor,” “in-body sensor,” and “ingestible sensor.” This search yielded a total of 96 authorized devices since 1982, after removing duplicate results. Note that this count includes various subcutaneous glucose sensors, which can also be defined as partially wearable sensors. Therefore, there are fewer than 96 fully implantable sensors approved by the FDA.

In part, this may be due to the challenges developers face during the planning, design, and implementation of these devices. Implantable sensors are highly complex devices that require advanced engineering and technical expertise to design and manufacture.^234^ Developing these devices involves multiple stages such as researching, prototyping, testing, and validating.^103^ This process can be challenging, and it can be difficult to transition from laboratory testing to real-world implementation. The complexity of these devices may also make them more susceptible to technical problems, which can further complicate their use in clinical settings.^235^ However, there are also a whole host of non-technical challenges facing implantable sensors that contribute even more to this disparity.

### A. Clinical challenges

The primary challenge to any clinical application is ensuring the safety and efficacy of the device. Implantable sensors are highly complex devices that require advanced engineering and technical expertise to design and manufacture.^234^ Developing these devices involves multiple stages including researching, prototyping, testing, and validating.^236^ This process can be daunting, and it can be difficult to transition from laboratory testing to real-world implementation. The complexity of these devices (cf. engineering) may also make them more susceptible to technical problems, which can further complicate their use in clinical settings.^235^

#### 1. Limited availability

Implanted sensors are not widely used in clinical practice, primarily due to their limited availability.^237–240^ Many medical centers lack the necessary infrastructure, resources, and expertise to offer these devices to their patients.^62,241^ The prevalence of implantable sensors varies across different healthcare systems and reimbursement policies, in that some countries have more favorable policies than others^62,242^

#### 2. Cost

**FIG. 9.**
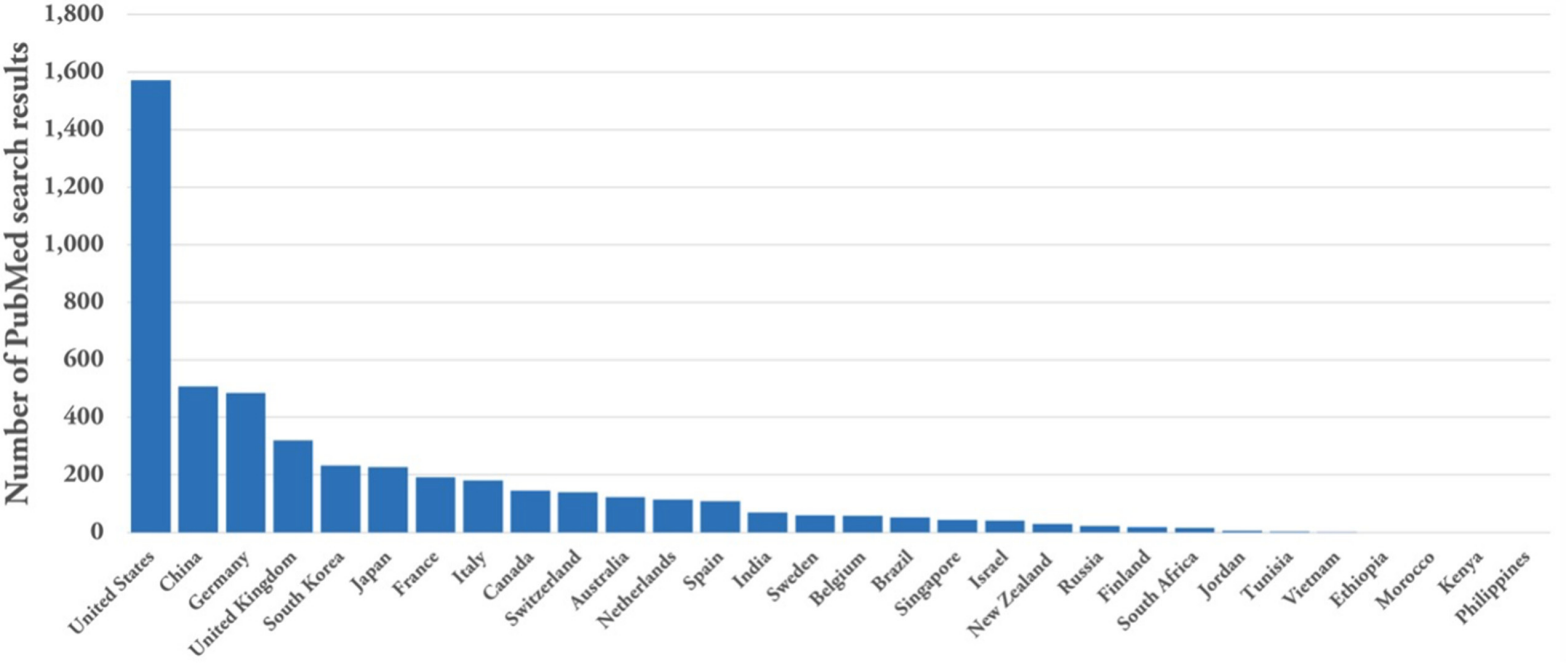
Distribution of PubMed results by country of affiliation for the search terms implantation and sensor. The bar chart shows that the technology related to implantation and sensors is mostly available in developed countries, whereas countries with fewer resources have limited access. These findings underscore the need for greater investment and support to increase accessibility and equity.

The high cost of implantable sensors can make them unaffordable for many patients and healthcare systems. Although they can be cost-effective in terms of the worldwide healthcare system, the cost per device of implanted sensors can be a substantial obstacle to their widespread clinical use.^243^ This can be incredibly challenging for patients with chronic conditions who may require long-term monitoring since the costs can add up over time. In comparison to wearable sensors, implanted sensors tend to have a higher cost due to the complexity of their design and the invasive nature of their implantation procedure. While wearables can be less expensive, they also have limitations in terms of accuracy and duration of use. The choice between wearable and implanted sensors ultimately depends on the individual's specific needs and the goals of the healthcare intervention. A search in PubMed by country of affiliation using the terms “implantation” and “sensor” (see Fig. 9) found that the US, China, Germany, the United Kingdom, South Korea, Japan, and France had the highest results for this search. This suggests that implantable sensor technology is not yet widely available in the developing nations, including countries such as Ethiopia, Morocco, Kenya, and the Philippines, which had 0 results in the search. One example of the United States' dominance in implantable sensor technology is the CardioMEMS device, which has been implanted in 550 patients across 64 centers in the country.^244^ Note that this search was restricted to MEDLINE (a biomedical bibliographic database) by selecting it from the subset menu.

The financial development of medical devices typically includes research and development (R&D) costs, regulatory costs, manufacturing costs, marketing and sales costs, and legal and patent costs. These costs can be high and are estimated to be over $100 million for some medical devices. According to an analytical cost model reported by Sertkaya *et al.*,^245^ the estimated mean expected capitalized development cost per therapeutic complex medical device in the United States is $522 million, most of which is devolved to the nonclinical development stage.

Due to these high development costs, only large medical device companies with considerable financial resources are typically able to bring these technologies to market. However, despite the high costs of development, these medical devices are often found to be cost-effective^243,246^ in the long run, making them a worthwhile investment.

#### 3. Ethical and legal concerns

Implantable sensors can raise ethical and legal concerns such as the requirement to obtain informed consent from the patient and ensure data privacy and security. In many countries, these issues are addressed through the Helsinki Declaration, a set of ethical principles for medical research involving human subjects. Physicians may also be cautious about utilizing these devices since they want to keep their patients “device-free,” consistent with the goal of maintaining the patient on the lowest number of prescriptions needed and preventing polypharmacy.

#### 4. Lack of real-world information

Although there is extensive literature reporting clinical trials and long-term clinical outcomes from implantable devices, there is a lack of real-world data on the application of implantable sensors in medicine. For instance, to date, orthopedic smart implants with sensing properties have been used exclusively as research tools.^235^ As outlined in the preceding sections, only a limited number of pressure sensing technologies have obtained FDA approval and undergone testing in real-world scenarios.^62^ This can make it difficult for physicians and healthcare organizations to make informed decisions about using these devices. While clinical trials and outcome data provide important information on the efficacy and safety of implantable devices, they do not necessarily reflect the challenges that clinicians and patients may face during the routine use of these devices in real-world settings.^247^ The lack of real-world evidence may make obtaining funding for these devices more complex, thus limiting their application in the clinical context. Despite these challenges, implanted sensors have several advantages over current tools. They provide continuous, real-time monitoring of a patient's physiological parameters, which can help detect and treat health problems early before they become more severe.^1^ Implanted sensors can reduce the need for frequent hospital visits and allow patients to live more independent lives.

### B. Patient-related challenges

In terms of the patient, it is crucial to consider how individuals perceive and adapt to the use of implanted sensors. A growing body of psychology research has explored how patients understand and accept these devices. For example, studies have shown that patients may experience varying levels of anxiety and discomfort before and after implantation and that the level of acceptance and satisfaction with the device can be influenced by factors such as the perceived benefits of the device and patients' level of control over its use.^91^ Studies have also found that factors such as patient education and ongoing support can play a critical role in helping patients feel more comfortable and confident about the use of implanted sensors.^92^ Overall, understanding the psychological and emotional aspects of patients' experiences with implanted sensors is critical to ensuring that the devices are used safely and effectively, and meet patients' needs and expectations.^250,251^

One of the main barriers to using implanted sensors is fear and anxiety. Patients may be concerned about the potential risks and complications associated with implantation,^252^ as well as whether the device will malfunction or fail. They may also have concerns about the long-term effects of having a foreign object in their body and the possibility of chronic pain or discomfort.^253^ The fear of being constantly monitored and the lack of privacy can also add to some patients' anxiety.^254^

Another barrier to using implanted sensors is the lack of understanding or familiarity with the technology. Some patients may be hesitant to use a device they do not fully understand or that they perceive as being too complex. Patients may also be concerned about the costs associated with the implantation and ongoing maintenance of the device, a concept known as “user burden,”^113^ especially if they do not have adequate insurance coverage. Another factor is social norms, where people may be concerned about how others perceive them when using an implanted device; they may feel that they will be stigmatized by others or will look different.^255,256^

In terms of physiological barriers, implanted sensors may cause trauma and discomfort during the implantation, and some patients may have allergic reactions to the materials used in the sensors.^257^ Some patients may have trouble with the healing process or develop an infection after implantation.^258^ The body's immune system may react to the device, resulting in a foreign body reaction^259^ in which the body attempts to remove or engulf it, which can cause the sensor to malfunction or fail. These issues may limit the longevity and effectiveness of the implanted sensor. Certain physiological conditions such as obesity, scar tissue, or underlying medical conditions may make implantation more difficult and increase the risk of complications.

Overall, implanted sensors have the potential to provide monitoring, identification, prevention, and restoration for various body systems. However, these devices also have psychological, social, and physiological barriers that can hinder their effectiveness and longevity. From the patient's perspective, it is essential to consider how individuals perceive and adapt to implanted sensors. Understanding the psychological and emotional aspects of patients' experiences with implanted sensors is crucial for ensuring that the devices are used safely and effectively and meet patients' needs and expectations. This suggests that medical and engineering communities need to work together to address these problems and improve the technology to make these devices more widely available, accessible, and patient-centric.

### C. Regulatory hurdles

Lack of specific and authorized protocols for bio-medical sensor implantation and management: the field is still relatively new and rapidly evolving, so there are no established standards for the implantation, calibration, or monitoring of these devices. This can lead to significant variations in clinical practice and may make it difficult for clinicians to compare results across different studies or institutions. The regulatory process for implantable sensors can be complex and time-consuming.^212^ The FDA must review and approve these devices before they can be used in clinical settings.^260^ This process can be lengthy and costly and constitutes a hurdle for small companies and researchers looking to develop and implement these devices. In addition, the guidelines for these devices change frequently, making it challenging for companies to navigate the regulatory process.^261^ To bypass regulatory difficulties, certain modern medical technologies are currently available as “open-source” or “do-it-yourself” and are supported by online communities.^262^ This concept draws on current FDA-approved technologies, although they are hacked and employed without FDA approval. As the need for implanted sensors grows, more open-source solutions may be produced as independent initiatives on the part of patients and healthcare professionals.^262,263^

The FDA has established classifications for approximately 1700 different generic types of medical devices, grouped into 16 medical specialties and assigned to one of three regulatory classes. The classification of a device is based on its intended use, risk level, and the level of control necessary to ensure its safety and effectiveness. Class I devices pose the lowest risk, and Class III devices pose the greatest. All classes of devices are subject to General Controls, which are the baseline requirements of the Food, Drug, and Cosmetic (FD&C) Act that apply to all medical devices. A premarket submission or application, such as a 510 K or premarket approval application (PMA), may be required depending on the class to which the device is assigned.^264^ Recently, President Biden signed legislation that no longer requires new medicines to be tested on animals to receive FDA approval. This change, long sought by animal welfare organizations, could mark a significant shift from animal use in drug safety regulation, which has been in place for over 80 years.^6^ Although this shift focuses on the approval of chemical compounds, it might point to future regulatory changes that may impact the development and implementation of medical devices and implantable sensors in clinical practice.

### D. Summary of challenges facing implantable sensors

In conclusion, discrepancy between the number of articles published on implantable sensors and their actual use in clinical practice can be attributed to a combination of factors, including the complexity of the devices, regulatory hurdles, high cost, patient and physician reluctance, and limited real-world data. Table VI presents a short checklist of factors to consider when designing implantable sensors. More research is needed to address these challenges and ensure the successful integration of implantable sensors into clinical practice. This research should address the practical challenges, such as developing simplified, cost-effective, and reliable implantable sensors, streamlining the regulatory process, and collecting real-world data on their safety and efficacy. This will help bridge the gap between laboratory testing and real-world implementation of implantable sensors and ensure their successful integration into clinical practice.

**TABLE VI.** Checklist of factors to consider when designing implantable sensors. The table lists the challenges, impact, and references for each challenge. These factors are crucial to ensuring the reliability, safety, and effectiveness of implantable sensors for medical applications.

Challenge	Description	Impact	References
Analytical sensitivity and selectivity	Ensuring accurate collection of information about biomarker	Reliable data for medical use and decision-making	106, 177, 181, 186
Biocompatibility and biofouling	Ensuring the materials do not harm the body, and vice versa	Patient comfort and health, as well as reliable and consistent performance over time	187, 191, 202
Localization, data transmission, and power	Facilitating optimal sensor function	Enabling new applications for implantable sensors	208, 212, 223
Lifetime and degradability	Ensuring sensor lifetime matches application	Reduced patient burden from replacement or extraction	223, 229, 230
Immunity to interference	Protecting the sensor from external interference that may affect data quality	High-quality data collection and transmission	61, 265
Data privacy	Ensuring secure data transmission and storage to protect patient privacy	Protection of sensitive medical information	252, 266
Ease of use	Making the technology user-friendly for patients and healthcare providers	Improved patient experience and physician adoption	267, 268
Limited real-world data	Lack of information on the long-term safety and efficacy of these devices in real-world settings	Difficulty obtaining funding and limited integration into clinical practice	248, 249
Patient and physician reluctance	Patients may have concerns about the device and the procedure, and physicians may be wary about utilizing them	Decreased willingness to use the technology	252
Complexity of device	Requires advanced engineering and technical expertise to design and manufacture	Increased risk of technical problems and difficulties with implementation	234–236
Regulatory hurdles	Complex and time-consuming approval process by the FDA, can be lengthy and costly, and guidelines frequently change	Barriers to adoption and difficulties navigating the regulatory process	212, 260, 261
High cost	Substantial cost per device and the cost of implantation and maintenance can be prohibitively high for patients and healthcare systems	Cost-prohibitive for many patients and healthcare systems	243–245

## VI. CHARTING A COURSE FOR THE FUTURE OF IMPLANTABLE SENSORS IN MEDICAL TECHNOLOGY

The development of implantable sensors is a complex process that requires collaboration between clinical and engineering professionals. In this review, we delved into the intricate relationship between clinical requirements and engineering challenges involved in developing implantable sensors. We provided a detailed examination of the obstacles that have prevented these sensors from being widely adopted in medical technology, including patient comfort and privacy concerns, and the need for minimal side effects. We also overviewed the clinical applications of implantable sensors and analyzed the types that were currently available, highlighting their advantages and disadvantages. By addressing these challenges and opportunities, this review thus provides a roadmap for allowing these emerging technologies to change the way we live.

Future research should explore the multiple aspects discussed here, such as long-term performance *in vivo* or enhancing the biocompatibility of devices through correct deployment and maintenance of materials and packaging, as well as finding new long-lasting power sources. Recent technological advances, such as the potential of AI to revolutionize multiple industries, the development of sophisticated algorithms, and better tissue integration will hopefully allow future implantable devices to follow the same path as pacemakers and cochlear implants in providing solutions for multiple medical conditions across multiple disciplines of medicine.

### A. Continuous monitoring

Continuous monitoring of physiological parameters through implantable sensors constitutes a revolutionary approach in the field of healthcare. This approach not only alters the interaction between these sensors and the human body but also their interaction with the external environment. The presence of sensors within the body, which constantly relay vital information, challenges conventional notions of communication and interaction with healthcare providers. This paradigm shift raises important questions that need to be addressed to fully leverage the potential of implantable sensors.^269^

Once implantable sensors are in place, healthcare providers can explore the possibility of remotely accessing and analyzing real-time data. This would enable physicians to monitor their patients' health remotely, allowing for proactive interventions and timely adjustments to treatment plans. For instance, a physician could remotely modify the sensor parameters based on uploaded real-time data, thus eliminating the need for the patient to physically visit the healthcare facility.^269,270^ Furthermore, the implementation of autonomously self-providing healthcare systems represents an intriguing avenue for exploration. These systems could autonomously monitor, report, and react to the data provided by implantable sensors. By integrating artificial intelligence algorithms, these systems could detect patterns, identify anomalies, and trigger appropriate responses, such as alerting healthcare professionals or initiating therapeutic interventions.^271^ The potential benefits of these self-providing systems are immense in terms of real-time and personalized healthcare that obviate the need for constant physical presence at medical facilities.^272^

### B. Integration of multiple implantable sensors

This review addressed different types of implantable sensors. While currently each sensor is independent, in the future, there may be an array of different sensors that communicate and form one holistic system that can provide constant information on the patient's physiology. While this is very appealing, the ability to create such a multiple sensor system is extremely challenging, since it requires constant communication and integration of information across modalities. Zhang *et al.*,^273^ who studied wearable sensors, identified several key obstacles to integrating multiple sensors: choosing the right sensor for each purpose, determining how many sensors are needed and their specific functionalities, and the intelligent and effective combination and processing of different data. This requires critical decision-making in terms of costs, recruitment, implementation, and data validity.^273,274^

### C. Autonomous implantable sensors

The full potential of implantable sensors lies not only with their ability to constantly monitor the physiological parameters of the body, or high sensitivity, but also the fact that these devices can trigger reactions to specific conditions in other implants, or in a sense act autonomously. Some systems that sense and respond have existed for years, such as pacemakers, insulin pumps, and DBS, but in recent years, the next generation of these autonomically implantable sensors has emerged. This review covers several advances, but many other innovations mentioned in the literature are currently at various stages of development. One of these, as discussed in Sec. III B, is the intriguing development of an implantable pressure sensor designed to regulate ICP and prevent potential nerve damage from fluid buildup.^160^ Feiner *et al.*^275^ showed how a hybrid system of living cells and electronics improved engineered cardiac tissue. They used a scaffold with embedded electrodes to monitor electrical signals, stimulate cell contractions, and synchronize tissue activity. This approach can potentially repair damaged cardiac conduction systems. The authors concluded that in the future, progress in hybrid systems could remotely monitor and regulate tissue function, inform physicians about patients' health, trigger regenerative processes, and create automated patches for better disease management.

By triggering the release of targeted therapeutic interventions such as drugs, electrical stimulation, or other therapies, the future of implantable sensors hold immense promise in preventing the progression of diseases and improving patient outcomes.^276^ Clearly, ensuring patient safety is paramount in the case of autonomous implantable sensors, including the controlled release of drugs in specific clinical situations to avoid potential harm. Therefore, it is crucial to develop robust verification protocols which confirm that therapeutic interventions are only initiated when medically necessary. These protocols minimize the risk of releasing drugs inappropriately or causing adverse effects, while preventing disease progression and improving patient outcomes.^277,278^ Medtronic's MiniMed™ 780G automated closed-loop insulin delivery system is a good example of prioritizing patient safety. This system allows the user to set a minimum target glucose level of 100 mg/dl,^279^ which has been proven effective in real-world settings.^280^ By maintaining glycemic control within a safe range, the system minimizes the risk of dangerous hypoglycemia, even if it means deviating from achieving more physiologically optimal glucose levels (between 80 and 90 mg/dl^281^). This example showcases the conscious decision to prioritize patient safety over the desire for an idealized outcome. Such considerations also tie into the crucial hurdle of obtaining regulatory approval for these novel autonomic sensor systems, which must conform to frameworks, such as the Active Implantable Medical Devices Directive (AIMD) 90/385/ECC^282^ developed by the Medicines and Healthcare Products Regulatory Agency. In the ever-changing landscape of healthcare policies, it is also critical to understand and meet the needs of healthcare providers such as government agencies and insurance companies, since these institutions influence the success of a product considerably.^283^ By planning compliance to the rules and regulations right from the start, it should be easier and more cost-effective to navigate the path to market access for new medical sensors.^277,278^

In conclusion, implanted sensors have immense promise in the world of medical technology. Through careful consideration of clinical requirements and engineering challenges, these obstacles can be overcome, and implantable sensors can become a widely adopted part of medical practice.

## Data Availability

The data that support the findings of this study are available from the corresponding authors upon reasonable request.
